# Phosphorescent cationic iridium(iii) complexes dynamically bound to cyclodextrin vesicles: applications in live cell imaging†
†Electronic supplementary information (ESI) available: Experimental procedures, absorption and emission spectra, isothermal titration calorimetry, cell culture experiments and synthesis. See DOI: 10.1039/c8sc02875c. The research data supporting this publication can be accessed at https://doi.org/10.17630/1254db92-71d2-45a6-ac87-247894110d66.


**DOI:** 10.1039/c8sc02875c

**Published:** 2018-08-09

**Authors:** Frauke Schibilla, Anna Holthenrich, Boyi Song, Anna Lívia Linard Matos, David Grill, Diego Rota Martir, Volker Gerke, Eli Zysman-Colman, Bart Jan Ravoo

**Affiliations:** a Organic Chemistry Institute and Center for Soft Nanoscience , Westfälische Wilhelms-Universität Münster , Correnstrasse 40 , 48149 Münster , Germany . Email: b.j.ravoo@uni-muenster.de; b Institute of Medical Biochemistry , Center for Molecular Biology of Inflammation , Cells-in-Motion Cluster of Excellence (EXC1003-CiM) , Westfälische Wilhelms-Universität Münster , Von-Esmarch-Strasse 56 , 48149 Münster , Germany; c Organic Semiconductor Centre , EaStCHEM School of Chemistry , University of St Andrews , St. Andrews , Fife KY16 9ST , UK . Email: eli.zysman-colman@st-andrews.ac.uk

## Abstract

We report cationic Ir(iii) complexes functionalized with adamantyl groups designed to bind to β-cyclodextrin vesicles (CDV) with high affinity (*K*_a_ = 1 × 10^6^ M^–1^). The emission of the complexes is tuned by changing the nature of the cyclometalating ligands. The host–guest adduct of CDV and Ir(iii) complexes shows increased and significantly blue-shifted emission due to the lower mobility of the Ir(iii)-complexes residing in the less polar environment of the vesicle surface. Ir(iii)-decorated CDV are efficiently taken up by cells and can be used in live cell imaging. The CDV act as carriers to transport the phosphorescent complexes into cells where they selectively stain mitochondria.

## Abstract 2

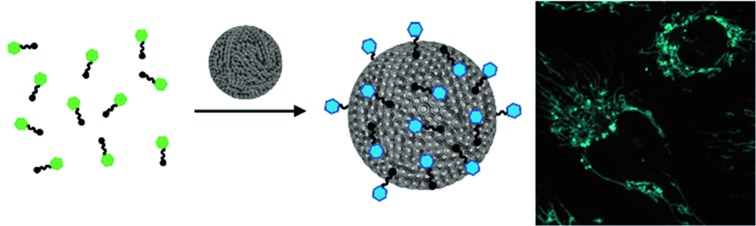
Cyclodextrin vesicles decorated with luminescent Ir(iii)-complexes are used as contrast agents for cell imaging.

## Introduction

Cationic Ir(iii) complexes containing two bidentate cyclometalating ligands (C^N, *e.g.*, 2-phenylpyridinato, ppy) and one bidentate diimine ligand (N^N, *e.g.*, 2-2′-bipyridine, bpy) are a family of phosphorescent materials featuring microsecond emission lifetimes (*τ*_PL_), high photoluminescence quantum yields (*Φ*_PL_), wide color tunability as a function of ligand design, and high photo- and chemostability.^1^,^2^ Cationic Ir(iii) complexes are widely used in organic light-emitting diodes (OLEDs),^3^–^5^ chemosensing systems^6^–^8^ and bioimaging.^9^–^11^ Bioimaging, in particular the visualization of specific processes and structures in live biological specimens such as cells, has greatly benefitted from the introduction of fluorescence-based imaging methods together with tools to deliver such fluorescence probes into cells. Luminophores used in live cell imaging should exhibit high chemoselectively, biocompatibility and optical brightness.^12^ Cationic Ir(iii)-complexes are attractive candidates for bioimaging, showing several advantages over organic fluorophores, due to their outstanding photophysical properties, including high *Φ*_PL_, photobleaching resistance due to both the long *τ*_PL_ and large Stokes shifts.^13^–^16^ Ir(iii)-alkyne probes, for example, were utilized for real-time imaging of dynamic processes in live cells by photoluminescence lifetime imaging.^17^ The cytotoxicity and solubility of probes are two major concerns for biological applications. One strategy to enhance the potential of cyclometalated Ir(iii) complexes as imaging (re)agents and cellular probes is to couple a poly(ethylene glycol) (PEG) chain resulting in good solubility and low cytotoxicity.^18^–^20^ For the purpose of photodynamic therapy a high cytotoxic activity can be useful, but for cellular probes minimal cytotoxicity is desired.^21^

Cyclodextrins (CD) are well known to form host–guest inclusion complexes with hydrophobic guest molecules,^22^–^24^ which are for example used for cell surface functionalisation^25^ or as nanocarriers.^26^ Amphiphilic CD derivatives self-assemble in aqueous solution to form CD vesicles (CDV).^27^ CDV are biocompatible and have, for example, been used as a model for biological cell membranes.^28^ The cavity of the CD in the CDV remains available for host–guest interactions. Thus, the surface of the CDV can be decorated by various functionalities *via* molecular anchors such as adamantane. These interactions have been exploited towards a “self-assembled glycocalyx”^29^,^30^ by decorating CDV with carbohydrates. Moreover, decoration of CDV with stimulus-responsive polymer shells resulted in nanocontainers that could be applied for triggered payload delivery into cells.^31^ However, CDV without polymer shells have never been used to deliver compounds into cells and CDV have never been functionalized with Ir(iii) complexes.

There are but a small number of studies that combine phosphorescent complexes with CDs.^32^ Yam *et al.* immobilized adamantane-functionalized Ru(ii) and Re(i) complexes on β-CD capped gold nanoparticles for the detection of biomolecules.^33^ Furthermore, a three component system consisting of a CD substituted Ir-complex as the photosensitizer, a viologen-based electron relay and CD-modified platinum nanoparticles as the catalyst was developed for the generation of hydrogen.^34^,^35^

**Fig. 1:**
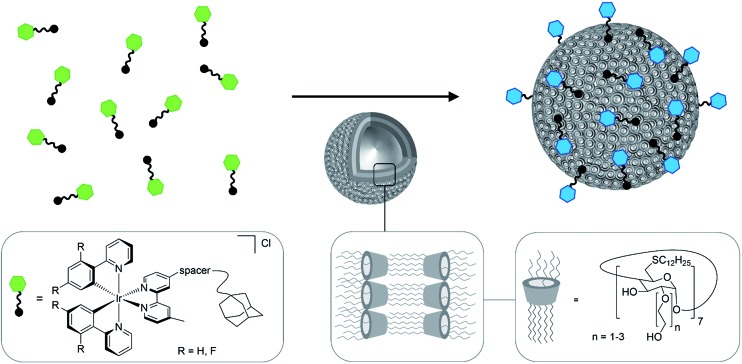
Schematic representation of the formation of luminescent vesicles by host–guest interaction of CDV and Ir(iii) complexes. The green and blue color represent the blue shift of the emission upon the immobilization of the Ir(iii) complexes on the CDV.

In this study, five water-soluble cationic Ir-complexes, four of which are functionalized with adamantyl units that can form host–guest assemblies with CDV, were generated and analyzed. The emission properties of the Ir-luminophores were tuned by using either 2-phenylpyridine (ppyH) or 2-(2,4-difluorophenyl) pyridine (dFppyH) as the cyclometalating ligands. The ancillary ligand constituted of a 2,2′-bipyridine (bpy) functionalized with either one or two tetraethylene glycol chains bearing a terminal adamantyl group (Fig. 1). The tetraethylene glycol linker was used to improve the water solubility, with the added benefit of lowering cytotoxicity. The Ir-decorated CDV were applied as luminescent probes in live cell imaging experiments allowing the visualization of intracellular compartments.

## Results and discussion

**Fig. 2:**
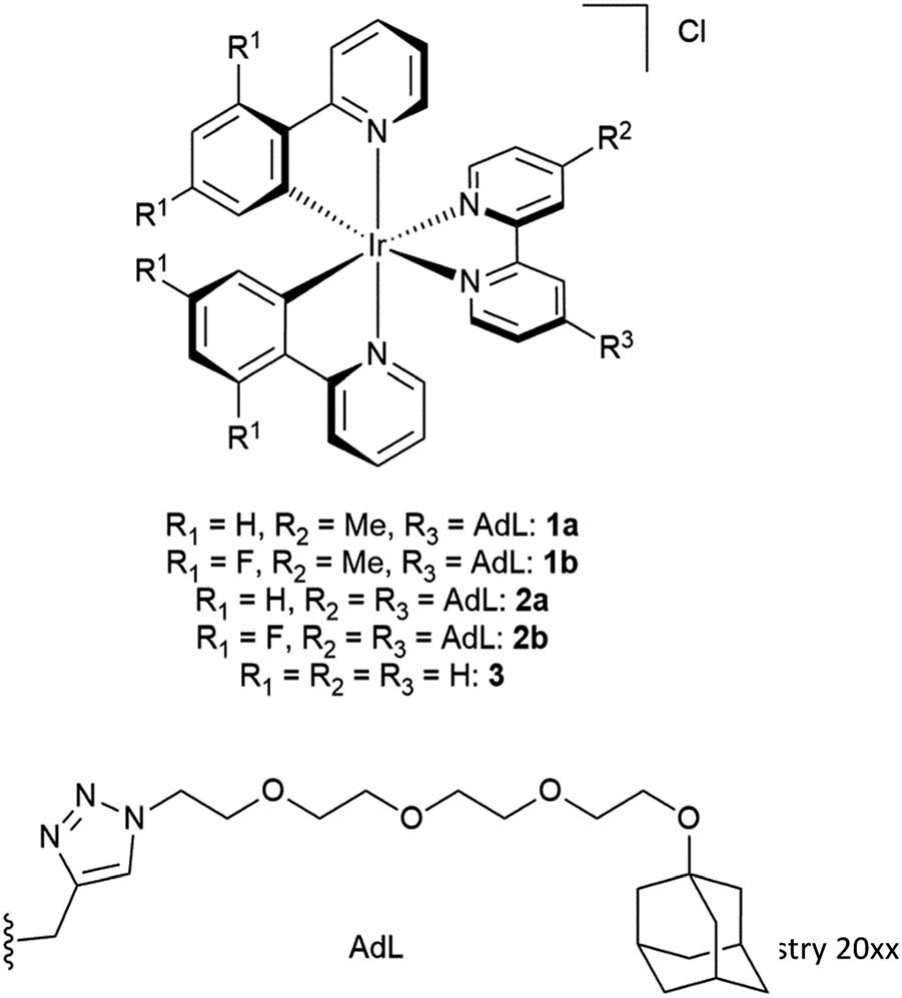
Molecular structure of Ir(iii) complexes **1a**, **1b**, **2a**, **2b** and **3**.

The investigated adamantane-functionalized Ir(iii)-complexes **1a**, **1b** (one adamantane substituent), **2a** and **2b** (two adamantane substituents) and reference compound [Ir(ppy)_2_(bpy)]Cl, **3**, are shown in Fig. 2. The synthesis is described in the ESI.†

### Photophysical characterization

The photophysical properties of the complexes were investigated by steady-state and time-resolved emission spectroscopy in both acetonitrile (MeCN) and phosphate buffer solutions, (PB; pH = 7.4). The photophysical properties of **1a** and **1b** are summarized in Table 1. All complexes mainly absorb in the UV region up to *ca.* 480 nm in both MeCN and PB, with the fluorinated complexes (**1b** and **2b**) showing a significant shoulder at around 300 nm (ESI, Fig. S1†). The high energy absorption bands above 300 nm can be assigned to spin-allowed π–π* (ligand-centered, LC) transitions while bands between 300 nm and 425 nm are associated with mixed of spin-allowed metal-to-ligand charge-transfer (^1^MLCT) and ligand-to-ligand charge-transfer (^1^LLCT) transitions.^36^ Complexes **1a**, **2a**, and **3** each show broad and unstructured orange emission in PB with an emission maximum at 565 nm. The presence of the triazole-tetraethylene glycol chains on the ancillary ligand has little effect on the optoelectronics of the complex. Complexes **1b** and **2b** show significantly more intense, broad and unstructured green emission at 525 nm in PB (ESI, Fig. S1†), which is the result of the inclusion of the electron-withdrawing fluorine atoms on the cyclometalating ligands.^37^

**Table 1:** Photophysical data of **1a** and **1b** in degassed (deg.) MeCN, deg. PB and PB (air)

Entry	*λ*_PL_ ^*a*^ [nm]	*Φ*_PL_ [%]	*τ*_PL_ ^*d*^ [ns]
**1a**, deg. MeCN	557	34.6^*b*^	475
**1a**, deg. PB	565	3.8^*b*^	74
**1a**, PB (air)	565	3.5^*b*^	73
**1b**, deg. MeCN	520	62.8^*c*^	1310
**1b**, deg. PB	525	34.2^*c*^	791
**1b**, PB (air)	525	14.8^*c*^	459

^*a*^ *λ*_exc_ = 360 nm.

^*b*^ *λ*_exc_ = 420 nm, using [Ru(bpy)_3_]Cl_2_ as the standard (*Φ*_PL_ = 4% in aerated H_2_O at 298 K).^38^

^*c*^ *λ*_exc_ = 360 nm, using quinine sulfate as the standard (*Φ*_PL_ = 54.6% in 0.5 M H_2_SO_4_ at 298 K).^39^

^*d*^ *λ*_exc_ = 378 nm.

Photoluminescence lifetimes, *τ*_PL_, and photoluminescence quantum yields, *Φ*_PL_, of **1a** and **1b** were measured in degassed MeCN, degassed PB, and PB in air. In MeCN, **1a** exhibited a *τ*_PL_ of 475 ns with a *Φ*_PL_ of 35%. For **1b** the *τ*_PL_ is considerable longer at 1310 ns, along with a correspondingly higher *Φ*_PL_ of 63%. In general, both Ir-complexes **1a** and **1b** show a blue-shifted emission and a higher *Φ*_PL_ compared to literature complexes [Ir(ppy)(bpy)]PF_6_ (ref. 40) or [Ir(dFppy)(bpy)]PF_6_,^41^ respectively. The emission was significantly quenched in aqueous solution, which was previously observed for other Ir-complexes.^42^–^44^ In PB the photophysical properties of **1a** did not show any sensitivity to O_2_ (∼73 ns, 4%, with and without air). On the other hand, **1b** did show oxygen sensitivity, with considerable quenching of both the *τ*_PL_ and the *Φ*_PL_ compared to degassed conditions (791 ns, 34% to 459 ns, 15%).

### Interaction with cyclodextrin vesicles

The interaction of **1a** and **1b** with β-CD was investigated by isothermal titration calorimetry (ITC). Both show a 1 : 1 stoichiometry and association constants of around 10^4^ M^–1^, which are comparable to those reported for similar adamantane/β-CD interactions.^45^ The association constant of the fluorinated complex **1b** (*K*_a_ = 5.8 × 10^3^ M^–1^) is a little lower compared to the non-fluorinated analog **1a** (*K*_a_ = 1.9 × 10^4^ M^–1^, ESI, Fig. S7†). The addition of unmodified β-CD to **1a** or **1b** does not influence either the absorption or luminescence profiles of the Ir-complex (ESI, Fig. S2†).

**Fig. 3:**
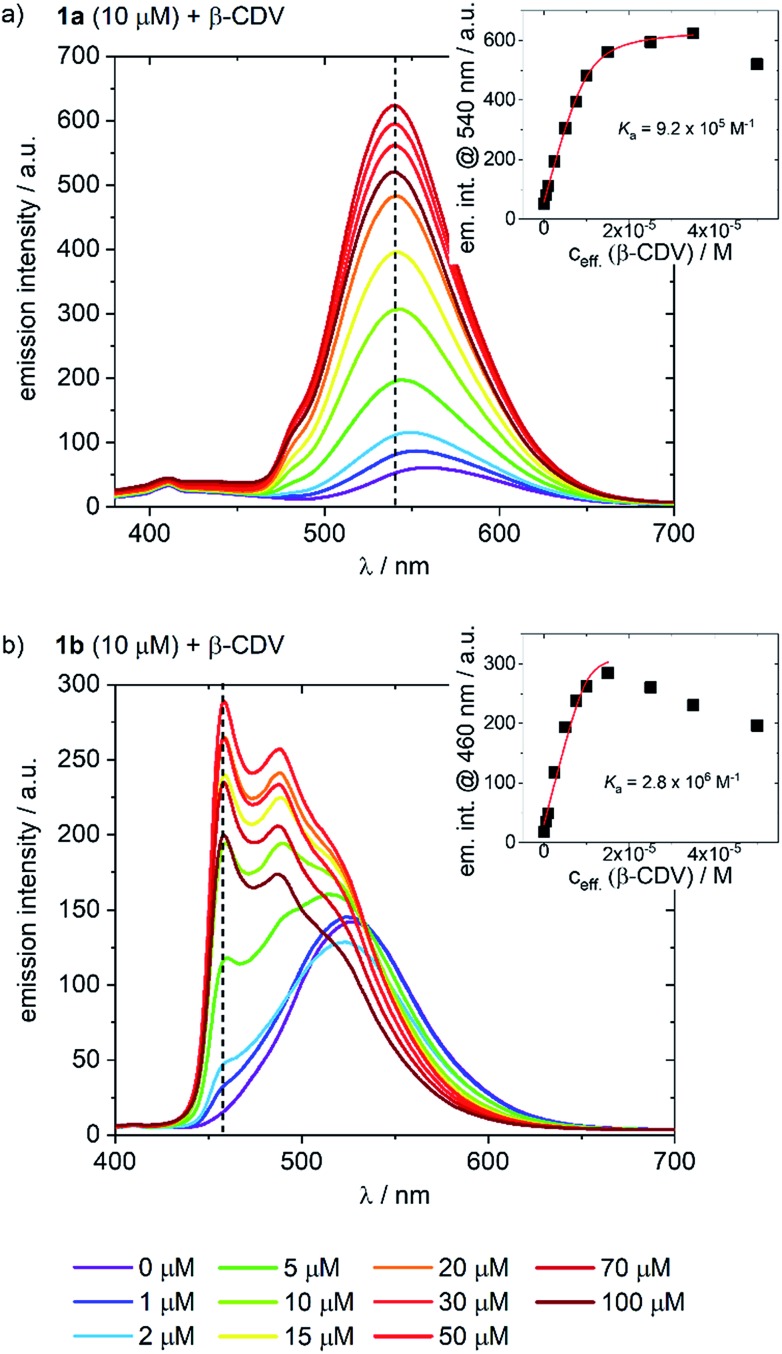
Emission spectra of (a) **1a** and (b) **1b** (10 μM) with increasing concentration of CDV (0–100 μM) in PB; *λ*_exc_ = 360 nm. The insets indicate the changes in emission intensity at (a) 540 nm and (b) 460 nm as a function of CDV concentration. Association constants (*K*_a_) were calculated using a Langmuir regression. The effective concentration of CDV (*c*_eff_) is used because it is assumed that only CDs on the outer surface of the CDV are available for host–guest interaction.

The behavior of the Ir-complexes with CDV was subsequently investigated. The preparation of the CDV is described in the ESI.† Titration of non-emissive CDV (1–100 μM) into a constant concentration of Ir-complex (10 μM) resulted in changes in both the emission intensity and profile (Fig. 3). Zeta-potential measurements of the vesicles confirmed the expected host–guest interaction. Initially, CDV showed a slightly negative zeta-potential of *ca.* –6.5 mV, which became positive (up to 11 mV) upon association with the Ir-complexes (ESI, Table S1†). With high CDV to Ir-complex ratios (∼5 : 1), the potential is expectedly lower, due to lower coverage of the vesicles by the Ir-complex. This positive surface potential confirms an adhesion of the Ir-complexes to the surface of the vesicles.

The addition of CDV to a PB solution of **1a** led to an increase in the emission intensity concomitant with a hypsochromic shift and a small shoulder appearing at 480 nm (Fig. 3a). At high CDV concentrations (>70 μM) the emission intensity decreased. This is likely the result of aggregation-induced quenching, which was confirmed by DLS measurements and by scattering observed in the UV/vis spectra (ESI, Fig. S3/S8†). CDV were likewise added to **1b** (Fig. 3b), resulting in a shift of the emission to higher energy. However, two sharp, non-shifting bands at 485 and 460 nm with increasing intensity were observed, which are the result of a change in the nature of the emission from ^3^CT to ^3^LC. Association constants of **1a** and **1b** to CDV were calculated using a Langmuir regression of the fluorescence titrations (**1a**@CDV: *K*_a_ = 9.2 × 10^5^ M^–1^; **1b**@β-CDV: *K*_a_ = 2.8 × 10^6^ M^–1^; Fig. 3, inset). The binding constants are around two orders of magnitude higher than for pure β-CD indicating high affinity binding to the CDV. We attribute the high binding constants to electrostatic interaction of the cationic Ir(iii)-complexes with the negatively charged surface of the CDV.

Most likely, the change in the nature of the emission, along with the blue shift, is a result of the less polar environment around the Ir-complexes when they are bound to the surface of the vesicles. Similar to **1a**, at high CDV concentrations (>30 μM) the emission intensity of **1b** decreased, likely due to aggregation-induced quenching. The behavior of the complexes in the presence of CDV was investigated further. In PB, both **1a** and **1b** emit *via* a mixed CT state that was assigned based on a positive solvatochromic behavior (ESI, Fig. S4†); in apolar PhMe, the emission becomes structured and more ligand-centered, with maxima that align to those observed during the addition of CDV to the complexes.

To assess the contribution of the host–guest inclusion complexation of CDV and **1a** or **1b** to the observed changes in the emission spectra, we studied the addition of β-CDV to **3**, which acts as a negative control. A small increase in intensity and a blue-shift was observed, but these effects were negligible compared to those observed using **1a** (ESI, Fig. S5†). Thus, the change in the emission properties observed for **1a** and **1b** can confidently be attributed to the host–guest interactions of the Ir-complexes with the CDV.

The emission lifetimes of the Ir-complexes were measured in the presence of CDV. In all cases the *τ*_PL_ behavior was found to be bi-exponential, with a first component of a magnitude reminiscent of the Ir-complex without CDV (**1a**: 73 ns/**1b**: 459 ns), and a second, longer component of around 530 ns for **1a** and 1100 ns for **1b**, attributed to **1a** and **1b** bound to the CDV. This behavior may be explained by the dynamic nature of the host–guest system in which the Ir-complexes exist both as bound adducts and freely dissociated in solution. After degassing, the second lifetime component of **1a** increased to around 630 ns while the first component stayed almost unchanged. In the case of **1b**, both lifetime components increased to around 500 ns and 1600 ns, respectively.

The addition of a second adamantane unit onto the ancillary ligand in **2a** and **2b** was expected to increase the association constant of the **2a**/**2b**-CDV adduct through multivalency and thus increase the stability of the luminescent CDV while decreasing the amount of unbound Ir-complex in solution. To this end, the emission behavior of **2a** and **2b** with CDV was investigated. Both complexes demonstrated a behavior similar to **1a** or **1b**, respectively, but aggregated at lower CDV concentrations (ESI, Fig. S6†), which can be explained by the undesired cross-linking of CDV through the Ir-complex.

### Cell experiments

**Fig. 4:**
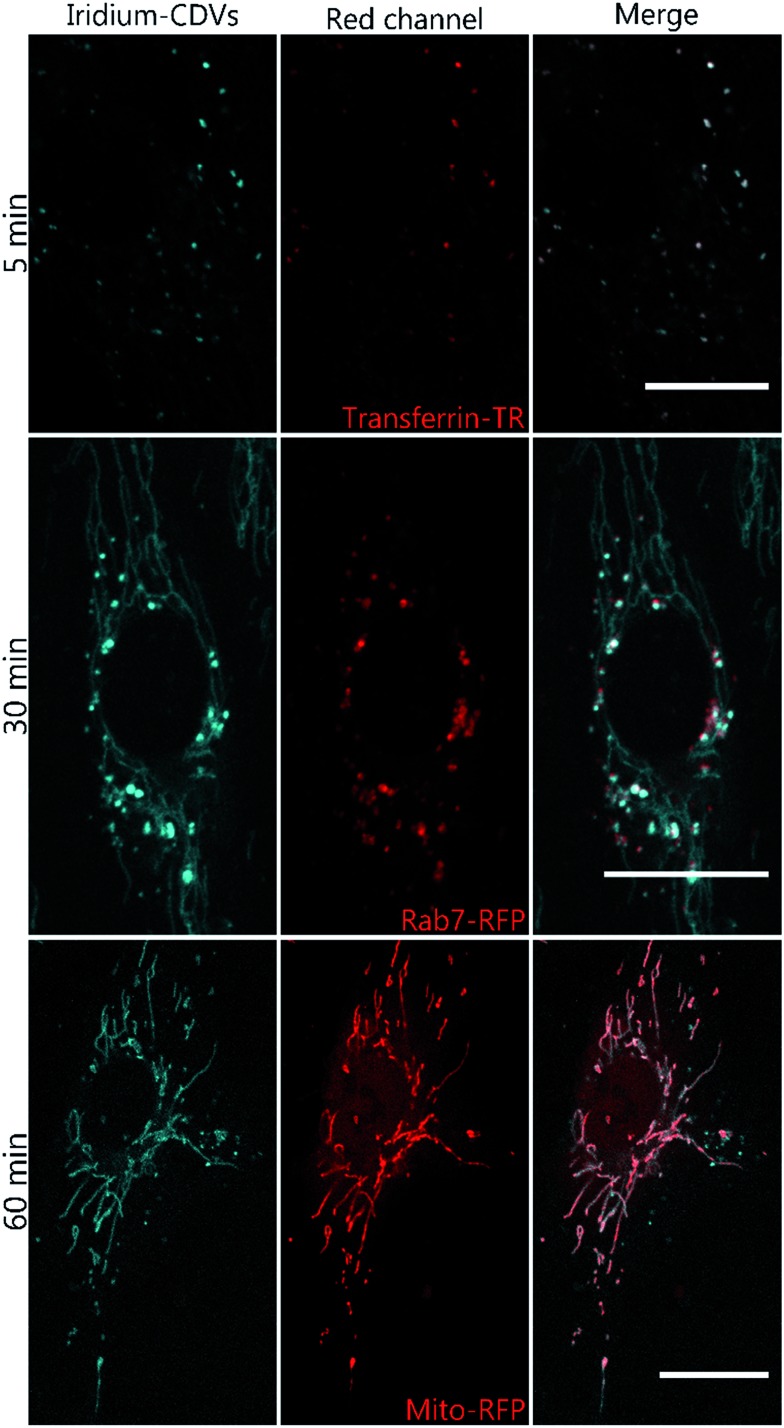
Live cell confocal microscopy images of HUVEC after incubation with **1b**-decorated CDV diluted in medium for 5 min, 30 min or 60 min. The complex is shown in blue. Cells were either transfected with Rab7-RFP (middle panel) Mito-RFP (lower panel) or pre-treated with transferrin-TexasRed (upper panel) for 5 min to label early endosomes. Scale bar 20 μm.

**Fig. 5:**
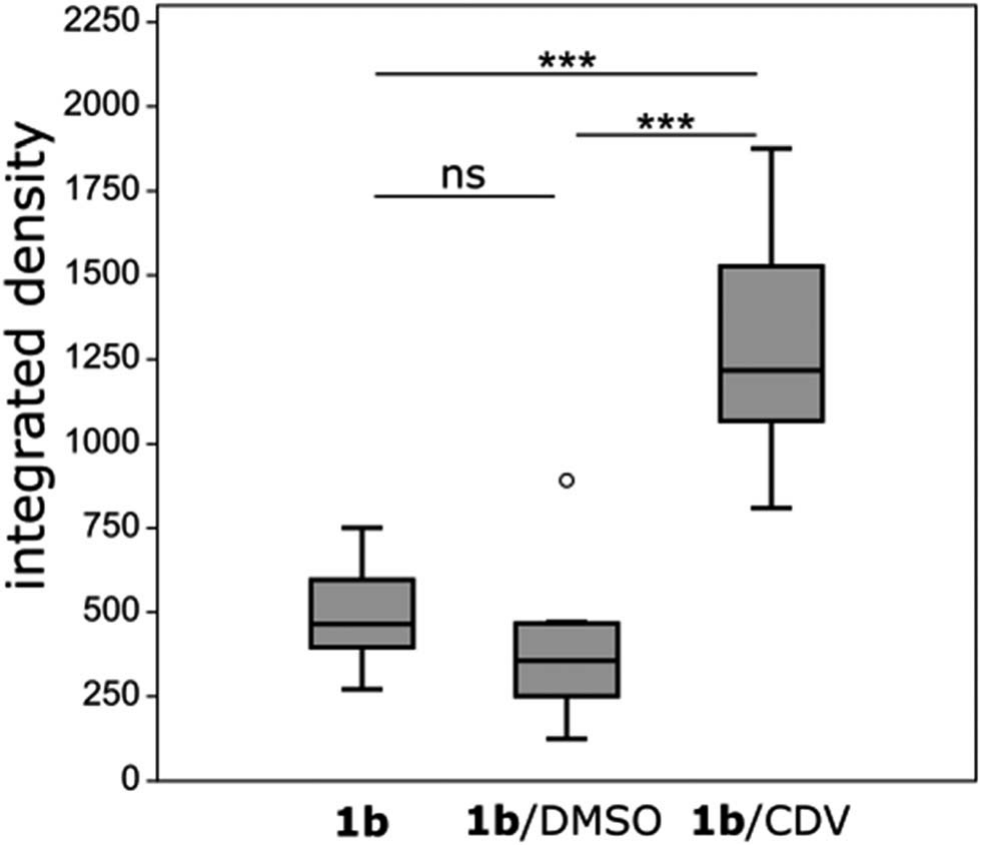
Analysis of cell uptake of **1b** after 45 min of incubation with either 1.25 μM **1b**, 1.25 μM **1b** and 1% DMSO or 1.25 μM **1b** and 3.75 μM CDV. HUVECs were imaged (*λ*_exc_ = 405 nm, *λ*_em_ = 410–556 nm) and analyzed by thresholding the fluorescence signal and calculating the integrated signal density (mean fluorescence value × area). *n* = 10 cells. ns = non statistically significant, *** = statistical significance, *o* = outlier data point. **1b**/**1b** +DMSO: *p* = 0.089; **1b**/**1b**@CDV: *p* = 0.0002; **1b** + DMSO/**1b**@CDV: *p* < 0.0001.

Ir-decorated CDV were applied as phosphorescent probes for live cell imaging, with the aim to develop a robust delivery system for imaging of intracellular compartments and trafficking. We selected complex **1b** for cell experiments, because this complex showed a higher emission intensity compared to **1a** in the *in vitro* studies. Human umbilical vein endothelial cells (HUVECs) were chosen as a model because they represent primary human cells. Toxicity and lytic activity of the Ir-complexes were analyzed by a cytotoxicity assay employing the compounds **1b**, **1b** in 1% DMSO, **1b**@CDV and only CDV at concentrations ranging from 0 to 100 μM on HUVECs. Release of lactate dehydrogenase as an indicator of cell lysis was only observed at concentrations exceeding 80 μM for CDV alone or **1b** alone. Likewise, **1b**@CDV showed relatively low cytotoxicity with significant cell lysing only observed at concentration above 15 μM. In contrast, **1b** dissolved in 1% DMSO was highly toxic inducing efficient cell lysis already at a concentration below 0.1 μM (ESI, Fig. S10†). Next, HUVECs were incubated with **1b**@CDV or **3** and CDV and subjected to live cell imaging (Fig. 4). Efficient cellular uptake of the Ir-complex was dependent on the presence of the adamantane unit, since **3** and CDV showed very little intracellular luminescence signal (ESI, Fig. S11†). Although both **1b** alone as well as **1b**@CDV were taken up and detected as elongated structures in the cell (ESI, Fig. S12†), **1b**@CDV showed significantly higher intracellular fluorescence signals (Fig. 5). To gain insight about the fate of the CDV after internalization, we tried to visualize the CDV uptake by labeling the CDV with rhodamine. After addition of **1b**@CDV-rhodamine to HUVECs, we were able to show the uptake of **1b** but we failed to detect any rhodamine signal (ESI, Fig. S13†). Thus, **1b** seems to disassociate from the CDV upon entering the cells. The CDV might fuse with the plasma membrane and laterally diffuse within the membrane, thus diluting the signal below the detection limit. The CDV might also act as a carrier, enhancing the solubility of **1b** in the aqueous medium. To further assess this, we tried to enhance uptake of **1b** by increasing its solubility through the addition of 1% DMSO. Uptake efficiency of **1b** in 1% DMSO was, however, low and comparable to the uptake of **1b** alone (Fig. 5).

To analyze the localization of the **1b**@CDV within cells, HUVECs were incubated with preformed **1b**@CDV (1.25 μM **1b** complex and 3.75 μM CDV) diluted in medium and imaged after 5, 10, 20, 30 and 60 min (ESI, Fig. S14/15/16†). Initial experiments indicated an uptake of the complex *via* small vesicles and a subsequent localization to structures possessing a morphology reminiscent of mitochondria. To verify this localization and to identify the cellular compartments accessed by **1b**, HUVECs were either transfected with a construct encoding the Mitochondrial Targeting Signal conjugated to mRFP (hereto referred as Mito-RFP) or the late endosomal marker Rab7-RFP. To label early endosomes, untransfected cells were incubated with 50 μg mL^–1^ Transferrin-TexasRed for 5 min before starting the uptake of the complex. A partial co-localization with transferrin can be observed 5 min past addition of **1b**@CDV (Fig. 4, ESI, Fig. S16†). After 10 min the Ir-complex starts to partially co-localize with Rab7 (ESI, Fig. S15†). This strongly indicates that **1b**@CDV is taken up *via* an endocytic pathway, where it remains during endosomal maturation to late endosomes. At around 30 min, co-localization between the Ir-complex and Rab7 is the strongest (Fig. 4). After 30 min, the Ir-complex also starts to co-localize with mitochondria, indicative of a transfer from late endosomes to the mitochondria (Fig. 4, ESI, Fig. S14†).

The specific association of **1b** with mitochondria that is even more evident at longer incubation times most likely is a consequence of both the high cationic charge and the lipophilicity of the Ir-complex, resulting in a preferred interaction with the mitochondria-specific lipid cardiolipin. The mitochondrial signal of **1b** increases over the observed time period of 90 min and remains for at least 24 h. It should be noted that Ir(iii) complexes were already used to stain mitochondria in live cell imaging and it was shown that Ir-complexes possess several advantages to conventional Mitotracker reagents:^46^–^50^ due to their photostability, Ir-complexes can be used in long time imaging of cells and as opposed to Mitotracker, their localization to mitochondria is not dependent upon the mitochondrial membrane potential. MTT assays conducted in HeLa cells showed that the used complexes possess little cytotoxicity. In our work, more sensitive primary cells (HUVECs) were used and, importantly, no cytotoxic effects were observed. Thus, the immobilization of Ir-complexes on CDV result in a highly soluble and less toxic imaging agent that demonstrates improved uptake into cells. Due to their versatile intrinsic properties, the easy chemical modification and modular construction, CDV represent a diverse class of delivery systems in cellular approaches.

## Conclusions

In summary, we have shown that two cationic Ir-complexes bearing adamantane anchoring units bind strongly to CDV giving rise to enhanced emissions that are shifted to higher energies compared to measurements in aqueous solution. This observation is attributed to a less polar and more viscous local environment near the CDV surface. Importantly, Ir-complexes immobilized on CDV can be readily internalized by cells where they eventually localize to mitochondria following transportation through the endocytic pathway. Ir-complexes without CDV were taken up with very little efficiency, demonstrating the capability of these vesicles as delivery vehicles. CDV decorated with luminescent Ir-complexes are therefore promising agents for bioimaging. We envisage that due to the modular self-assembly strategy, CDV can be developed into versatile platforms for theranostic applications.

## Conflicts of interest

There are no conflicts to declare.

## Supplementary Material

Supplementary information
