# Supplementary material for: Phosphorescent cationic iridium(iii) complexes dynamically bound to cyclodextrin vesicles: applications in live cell imaging
Source: Chem Sci. 2018 Aug 9;9(40):7822–8. doi: 10.1039/c8sc02875c (PMC6194495; doi:10.1039/c8sc02875c)
Supplement: Supplementary file 1 [file SC-009-C8SC02875C-s001.pdf]

Electronic Supplementary Material (ESI) for Chemical Science.
This journal is © The Royal Society of Chemistry 2018

Electronic Supplementary Information

Phosphorescent Cationic Iridium(III) Complexes Dynamically Bound to Cyclodextrin Vesicles: Applications in Live Cell Imaging

Frauke Schibilla,[a] Anna Holthenrich,[b] Boyi Song,[c] Anna Lívia Linard Matos,[b] David Grill,[b] Diego Rota Martir,[c] Volker Gerke,[b] Eli Zysman-Colman*[c] and Bart Jan Ravoo*[a]

[a] Organic Chemistry Institute and Center for Soft Nanoscience, Westfälische Wilhelms-Universität Münster, Correnstrasse 40, 48149 Münster (Germany), E-mail: b.j.ravoo@uni-muenster.de

[b] Institute of Medical Biochemistry, Center for Molecular Biology of Inflammation, Cells-in-Motion Cluster of Excellence (EXC1003-CiM), Westfälische Wilhelms-Universität Münster, Von-Esmarch-Strasse 56, 48149 Münster (Germany).

[c] Organic Semiconductor Centre, EaStCHEM School of Chemistry, University of St Andrews, St. Andrews, Fife KY16 9ST, UK, E-mail: eli.zysman-colman@st-andrews.ac.uk

Experimental Procedures

Mass spectrometry: Electrospray ionization (ESI) mass spectra were recorded on a *MicroTof* (*Bruker Daltonics*) and nanospray ionization (NSI) mass spectra on a LTQ Orbitrap XL (ThermoFischer Scientific).

NMR: NMR spectra were recorded on a *Bruker AV 300*, *AV 400, AVII400, AVIII500 and AVIII HD500* (*Bruker Corporation*). Measurements were performed in deuterated solvents at room temperature. *MestReNova 11.0* (*Mestrelab Research S. L.*) was used for data analysis. The chemical shifts are given in parts per million (ppm) relative to the residual solvent signals. The multiplicity of the signals is labeled as singlet (s), douplet (d), triplet (t), quartet (q), heptet (h), multiplet (m) and broad (br) and the coupling constant *J* is noted in Hz.

Photophysical characterization: Absorption spectra were measured on a *V-650 UV-Vis double-beam* spectrophotometer (*JASCO Labor- und Datentechnik GmbH*) and fluorescence spectra were recorded on a FP-6500 spectrofluorometer (*JASCO Labor- und Datentechnik GmbH*) with an excitation wavelength of 360 nm. Disposable PMMA cuvettes (*BRAND GmbH & Co. KG*) were used for all measurements in aqueous solution and high precision cells made of SUPRASIL quartz glass (Hellma Analytics) were used for all measurements in organic solvents. The path length is 1 cm. The software *Spectra Manager Version 2* (*JASCO Labor- und Datentechnik GmbH*, Gross-Umstadt, Germany) was used for the operation of the spectrometer and data analysis.

Additionally, steady-state emission, excitation spectra and time-resolved emission spectra were recorded at 298 K using an fluoroSENS fluorimeter (Gilden Photonics Ltd, excitation source 150 W continuous Xenon arc lamp) and F980 spectrometer (Edinburgh Instruments Ltd.). All samples for steady-state measurements were excited at 360 or 420 nm, while samples for time-resolved measurements were excited at 378 nm using a PDL 800-D pulsed diode laser. Emission quantum yields were determined using the optically dilute method.[1] A stock solution with absorbance of *ca*. 0.5 was prepared and then four dilutions were prepared with dilution factors between 2 and 20 to obtain solutions with absorbances of *ca*. 0.1 0.075, 0.05 and 0.025, respectively. The Lambert-Beer law was found to be linear at the concentrations of these solutions. The emission spectra were measured. For each sample, linearity between absorption and emission intensity was verified through linear regression analysis and additional measurements were acquired until the Pearson regression factor (R^2) for the linear fit of the data set surpassed 0.9. Individual relative quantum yield values were calculated for each solution and the values reported represent the slope value. The equation $\Phi_s = \Phi_r(A_r/A_s)(I_s/I_r)(n_s/n_r)^2$ was used to calculate the relative quantum yield of each of the sample, where Φ_r is the absolute quantum yield of the reference, *n* is the refractive index of the solvent, *A* is the absorbance at the excitation wavelength, and *I* is the integrated area under the corrected emission curve. The subscripts s and r refer to the sample and reference, respectively. A solution of quinine sulfate in 0.5 M H_2SO_4 (Φ_r = 54.6%)[2] and $[Ru(bpy)_3]Cl_2$ in aerated H_2O (Φ_r = 4%)[3] at 298 K was used as external references.[4] Degassing of the samples was done by bubbling nitrogen through the solutions for 10 min. before measuring.

Isothermal titration calorimetry (ITC): ITC was performed with a *TA Instruments Nano ITC Low Volume* (*TA Instruments*) with a cell volume of 170 µL, 20 injections of 2.5 µL at 25 °C and a stirring rate of 350 rpm. The device was controlled by *ITCRun Version 2.1.7.0 Firmware version 1.31* (*TA Instruments, Waters Corp.*). The measured data were corrected by subtraction of a dilution measurement of the titrated compound to pure solvent and data analysis was done with *NanoAnalyze Data Analysis version 3.6.0* (*TA Instruments*, *Waters Corp*.).

Dynamic light scattering (DLS) and Zeta-potential measurements: Measurements were performed on a *Nano ZS Zetasizer* (*Malvern Instruments Ltd.*) with disposable *semi-micro PMMA* cuvettes (*BRAND GmbH & Co. KG*) with a path length of 1 cm or disposable DTS 1060 capillary cells (*Malvern Instruments Ltd*.) at 25 °C. The software *Zetasizer 7.11* (*Malvern Instruments Ltd.*) was used for the operation of the spectrometer and data analysis.

Preparation of vesicles: Heptakis[6-deoxy-6-dodecylthio-2-oligo(ethylene oxide)]-ß-cyclodextrin was synthesized as described before with ca. 14 ethylene oxide units.[5] 1000 µL of a 1.0 mM solution of amphiphilic ß-CD in chloroform were added to a round bottom flask. The solvent was removed by a stream of argon to yield a thin film. The solvent was completely removed under high vacuum for 5 min. Phosphate buffer solution (*p*H 7.4, 10 mM, 5 mL) was added and stirred overnight. The resulting suspension was sonicated for 15 min. Afterwards the suspension was extruded with a *LiposoFast* manual extruder (AVESTIN Europe GmbH) by repeatedly passing it through a polycarbonate membrane (100 nm pore size). DLS measurements showed an average hydrodynamic diameter around 160 nm.

Cell culture and transfection: Primary human umbilical vein endothelial cells (HUVECs), purchased from PromoCell (C-12203), were cultured at 37°C and 5 % CO_2 for up to 5 passages on CellBIND plates (Corning, CLS3296-40EA) in HUVEC mix medium consisting of Endothelial Cell Growth Medium 2 (ECGM2, PromoCell, C-22011) and M199 medium containing 10% fetal calf serum (FCS) (Sigma, F7524) at a 1:1 ratio. The medium was further supplemented with 30 µg/ml gentamycin (Sigma, G1397) and 15 ng/ml amphotericin B (Biochrom, A2612). According to the manufacture's instruction, HUVECs were transfected with 1-2 µg plasmid DNA per 10 cm^2 nearly confluent cells using the Amaxa Nucleofection kit (HUVEC Nucleofector Kit, Lonza VPB-

1002). Following transfection cells were cultured for 24 h in µ-Slide 2 Well Glass Bottom (Ibidi, 80287) pre-coated with collagen (Type I). Transferrin conjugated to TexasRed was ordered from ThermoFisher (T2865).

Mammalian expression vectors: The mRFP-Mito construct was obtained by exchanging the fluorescent tag of pmTurquoise2-Mito with mRFP from mRFP-C1 using restriction enzymes AgeI (NEB, R3552L) and XhoI (NEB, R0146L). mRFP1-C1 was a gift from Robert Campbell, Michael Davidson and Roger Tsien (Addgene plasmid # 54764). pmTurquoise2-Mito was a gift from Dorus Gadella (Addgene plasmid # 36208).[6] mRFP-Rab7 was a gift from Ari Helenius (Addgene plasmid # 14436).[7]

Live cell microscopy: Live-cell imaging employed a LSM 780 confocal laser scanning microscope (CLSM, Carl Zeiss) equipped with the objective lense Plan-Apochromat x 63/1.4 oil and differential interface contrast objective lenses (Carl Zeiss) in a 37°C environment with 5 % CO_2. Data was processed using Fiji.[8] For analysis of uptake efficiency, confocal pictures of single cells were thresholded for excitation at 405 nm (emission collected between 410 and 556 nm) and the integrated signal density (mean fluorescence value * area) was calculated for the thresholded area. Statistics were done using the Mann-Whitney U test. Artificial colors (cyan and red) were used for presentation of the data.

Uptake experiments: For uptake experiments, freshly prepared Ir-complexes and CDV in PBS (5 µM and 15 µM respectively) were diluted in mixed medium to final concentrations of 1.25 µM and 3.75 µM, respectively, and added to HUVECs. Uptake of the complex was monitored for 90 min in a live cell experimental setup (37°C and 5 % CO_2) using CLSM.

Cytotoxicity assay: Toxicity of CDV, **1b** and **1b**@CDV pre-solved in assay medium or **1b** dissolved in DMSO on HUVECs was assessed by measuring the lactate dehydrogenase (LDH) activity released from damaged cells. The cells were seeded in 96-well plates with density of 1500 cells/well and cultured for 2 d. Subsequently, the culture medium was replaced with assay medium containing different concentrations, of the compounds ranging from 0 to 100 µM. Thereafter, the plates were left in the incubator for 2h at 37°C. The maximal LDH activity that represents the control values was determined by addition of 1% Triton X-100 for 10 min to completely lyse the cells. After transfer of the cell supernatants to a new 96-well plate, they were mixed in a volume ratio of 1:1 with reaction mixtures containing the tetrazolium salt, 2-(4-iodophenyl)-3-(4-nitrophenyl)-5-phenyl-2H-tetrazolium (INT), followed by incubation for exact 30 min at 37 C by protected from light. The reaction was stopped by addition of HCl (1 N) to each well. The values of relative toxicity (percentage of LDH activity as compared to the maximal value obtained after Triton treatment) were calculated employing the equation 1 after measuring the absorbance at 492 nm on a Microplate Reader. The concentration-response curve obtained was fitted to a growth-sigmoidal function. LDH assays were carried out for each sample in sextuplicate (n = 6).

$$Cytotoxicity\ (\%) = \frac{Absorbance_{492}\ (sample) - Absorbance_{492}\ (low\ control)}{Absorbance_{492}\ (high\ control) - Absorbance_{492}\ (low\ control)} * 100 \qquad (1)$$

Supplementary Figures

Absorption and emission spectra:

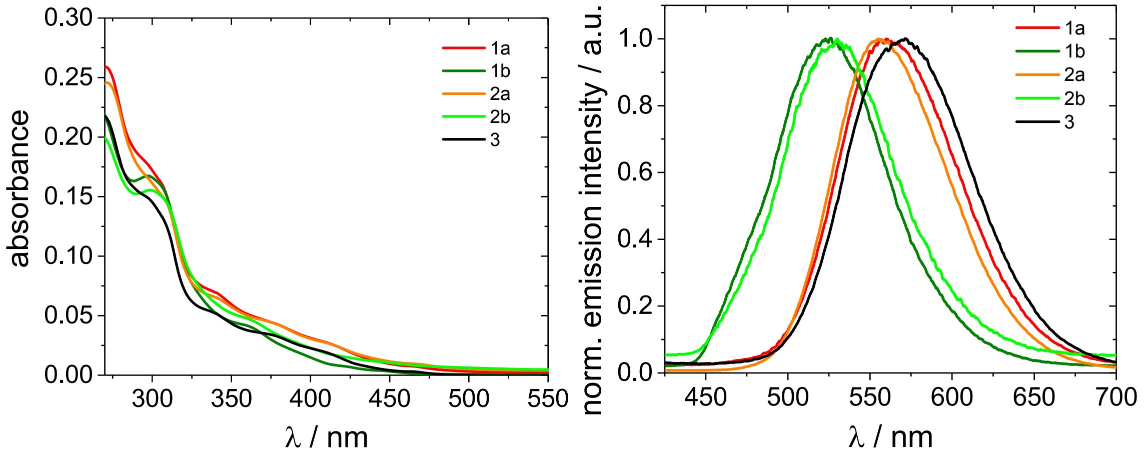

Figure S1: Absorption and emission spectra of **1a**, **1b**, **2a**, **2b** and **3** in phosphate buffer (pH = 7.4); λ_{exc} = 360 nm.

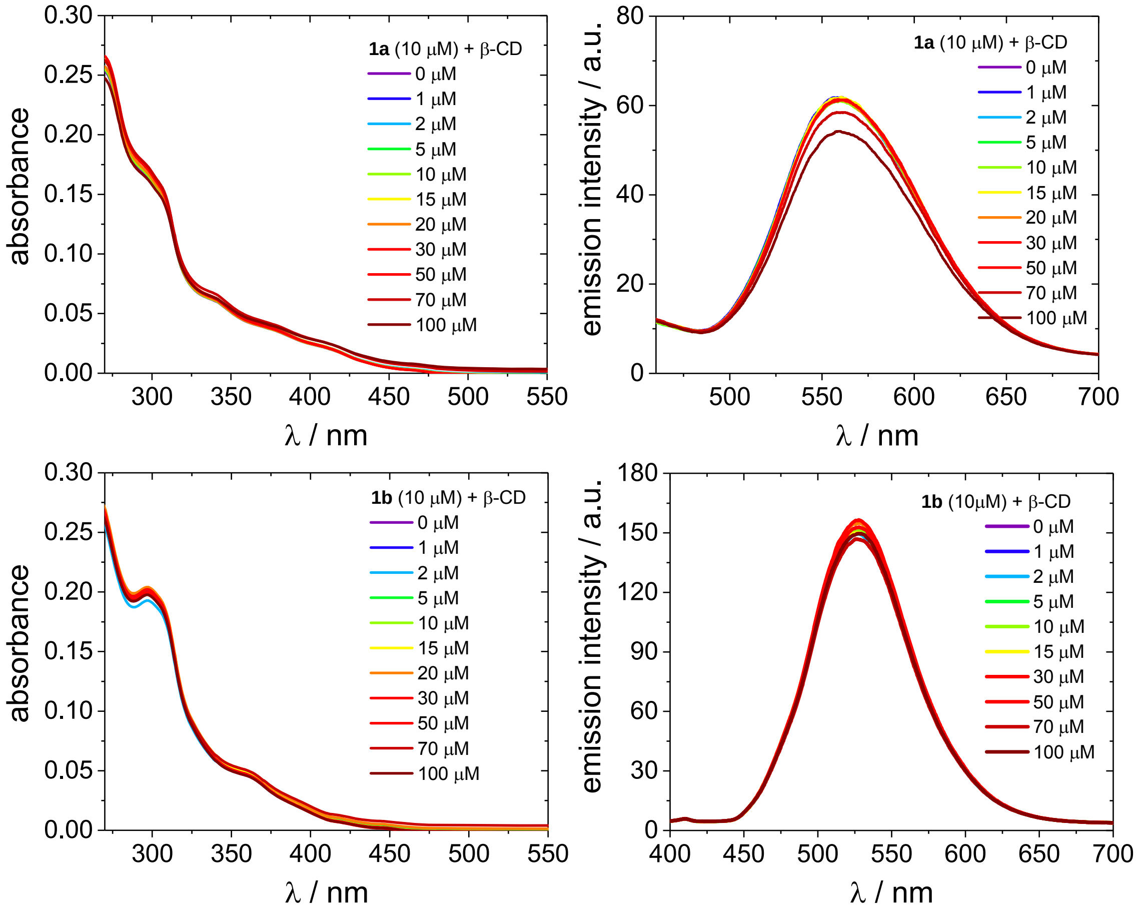

Figure S2: Absorption and emission spectra of **1a** and **1b** (10 μM) with different concentrations of β-CD (0-100 μM) in phosphate buffer (pH = 7.4); λ_{exc} = 360 nm.

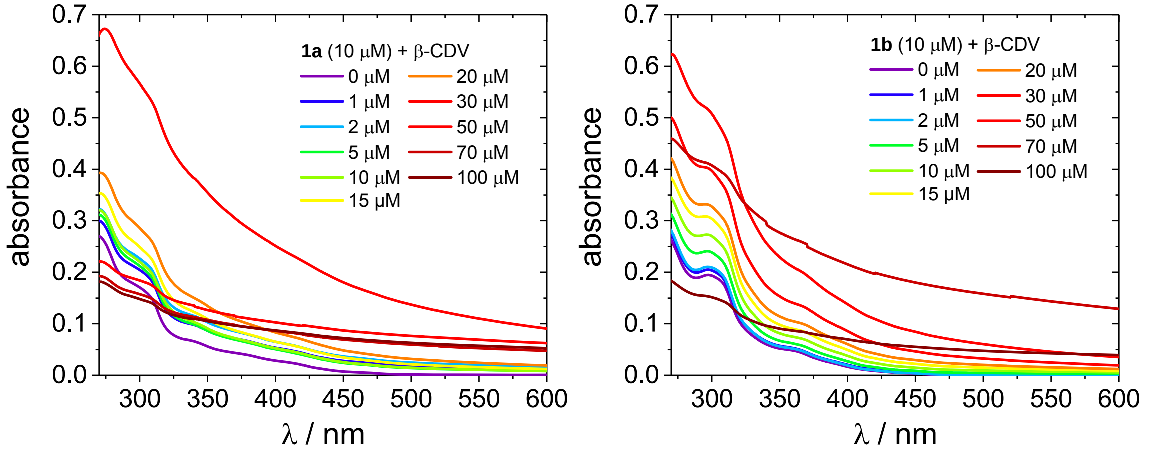

Figure S3: Absorption spectra of **1a** and **1b** with different concentrations of β-CDV (0-100 μM) in phosphate buffer (pH = 7.4).

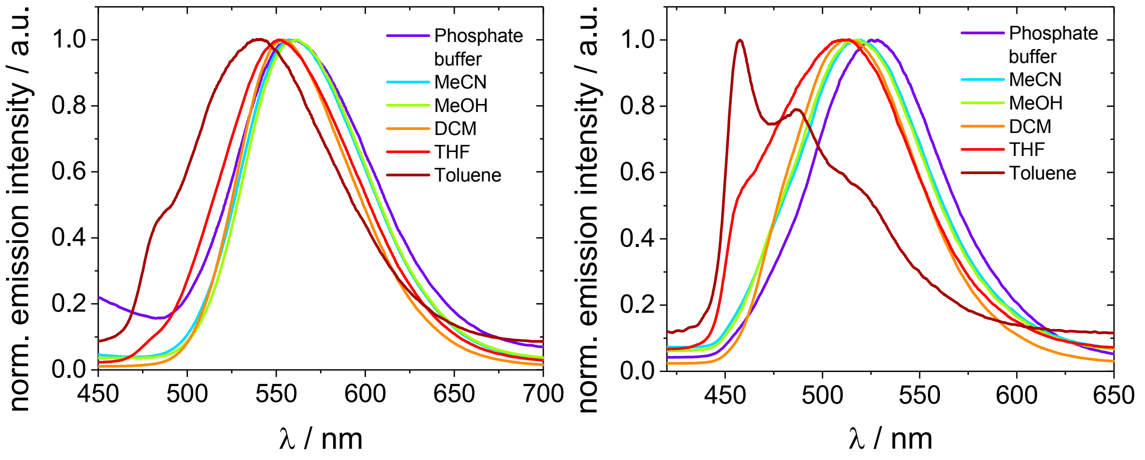

Figure S4: Normalized emission spectra of **1a** and **1b** in different solvents; λ_{ex} = 360 nm.

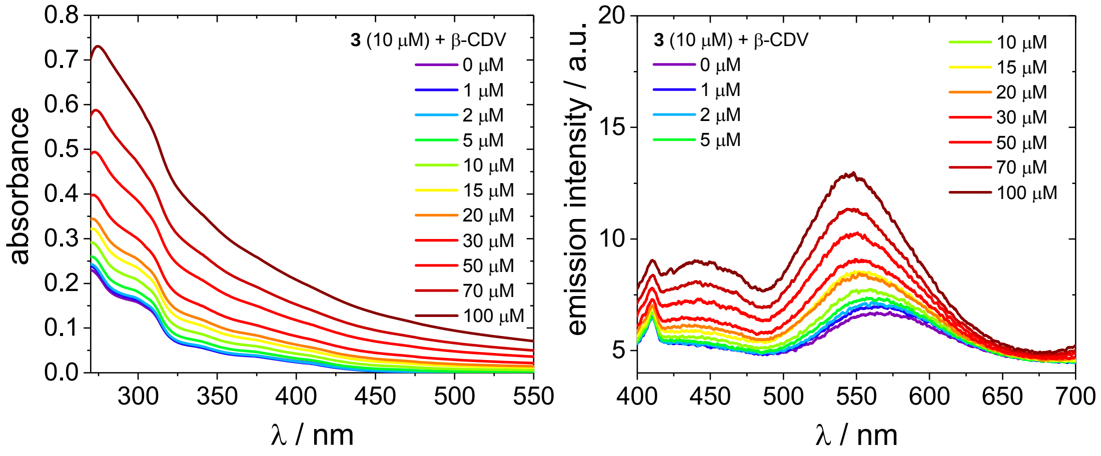

Figure S5: Absorption and emission spectra of **3** with different concentrations of β-CDV (0-100 μM) in phosphate buffer (pH = 7.4); λ_{exc} = 360 nm.

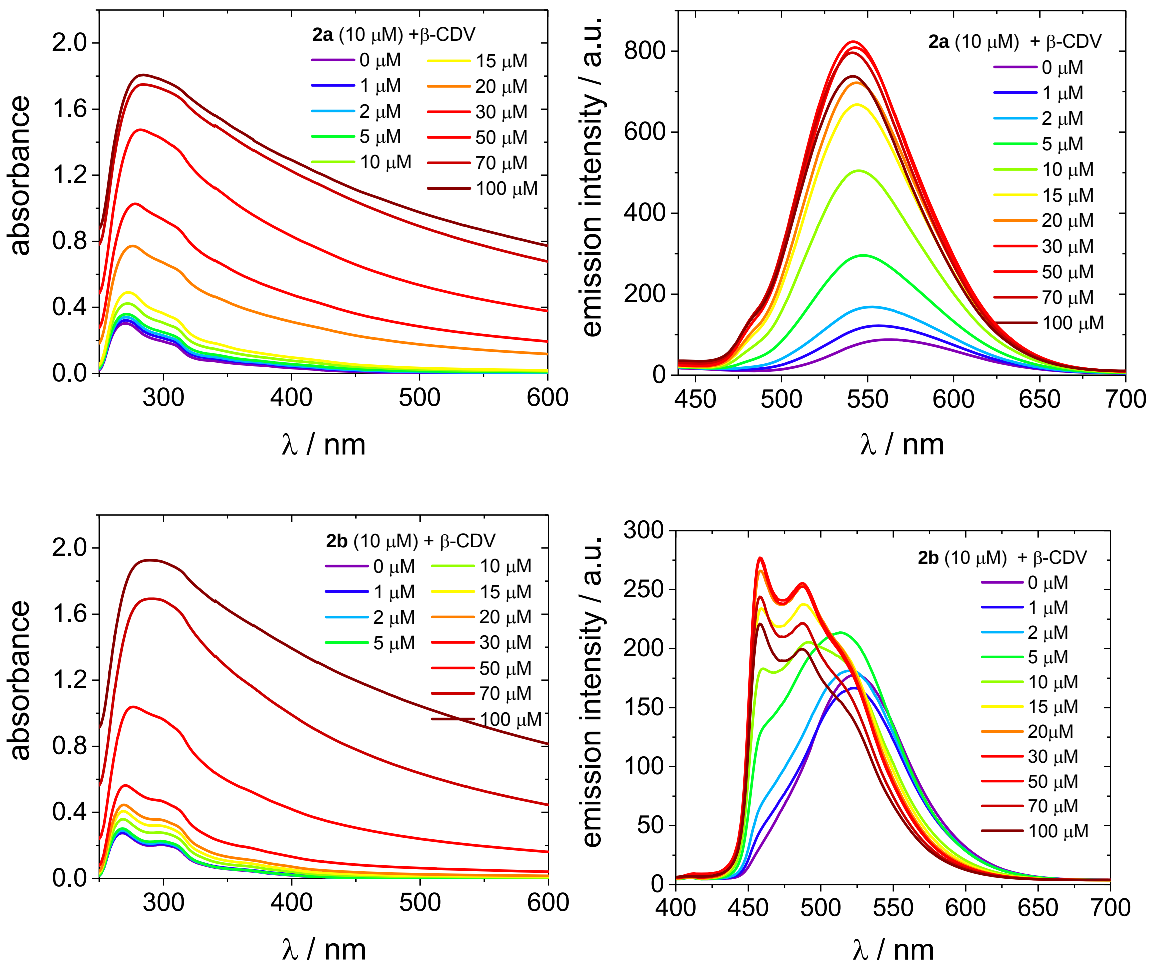

Figure S6: Absorption and emission spectra of **2a** and **2b** (10 μM) with different concentrations of β-CDV (0-100 μM) in phosphate buffer (pH = 7.4); λ_{exc} = 360 nm.

Isothermal titration calorimetry (ITC):

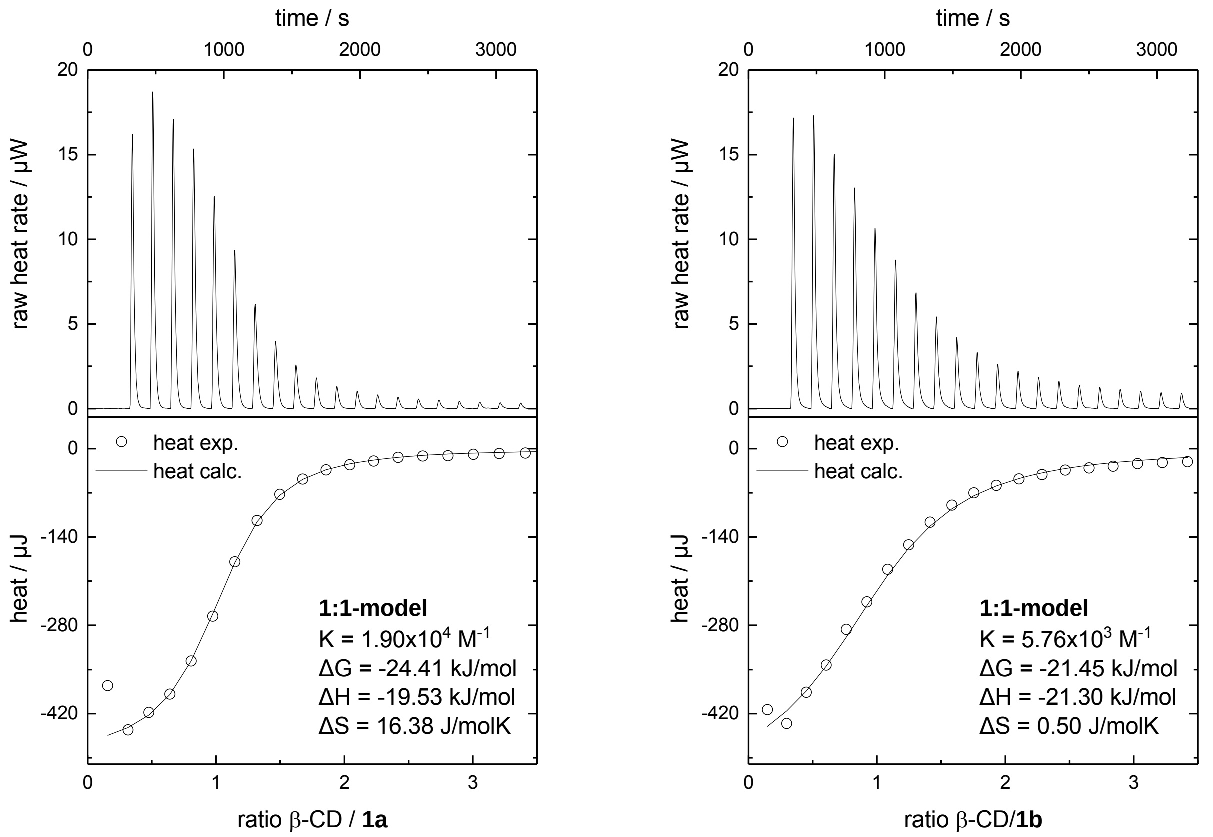

Figure S7: Isothermal titration calorimetry curve and corresponding fit of **1a** (1 mM) (left) and **1b** (1 mM) (right) with β-CD (10 mM) in H_2O and thermodynamic parameters for a 1:1 model.

Zeta-potential:

Table S1: Zeta-potential of β-CDVs with **1a** or **1b** respectively in PB at varying CDV concentration and fixed Ir-complex concentration (10 μM).

c (CDV) [μM]	Zeta-potential (**1a**) [mV]	Zeta-potential (**1b**) [mV]
5	9.55 ± 0.29	7.41 ± 0.42
10	9.87 ± 0.74	10.26 ± 1.61
15	9.92 ± 0.15	11.73 ± 0.74
20	10.87 ± 0.17	11.23 ± 0.63
30	8.84 ± 0.01	11.97 ± 1.59
50	3.57 ± 0.31	10.77 ± 1.17
70	1.92 ± 1.38	7.68 ± 1.03
100	-2.92 ± 0.15	0.73 ± 0.87

DLS measurements:

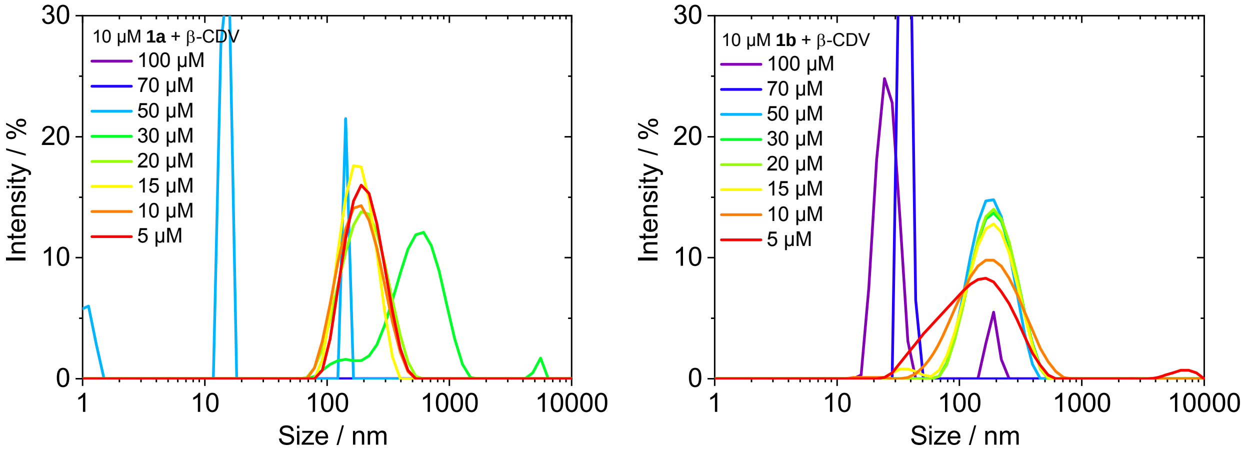

Figure S8: DLS measurements of **1a** and **1b** (10 μM) with different concentrations of CDV (5-100 μM) in phosphate buffer (pH = 7.4).

Photographs of CDV decorated with luminescent Ir-complexes:

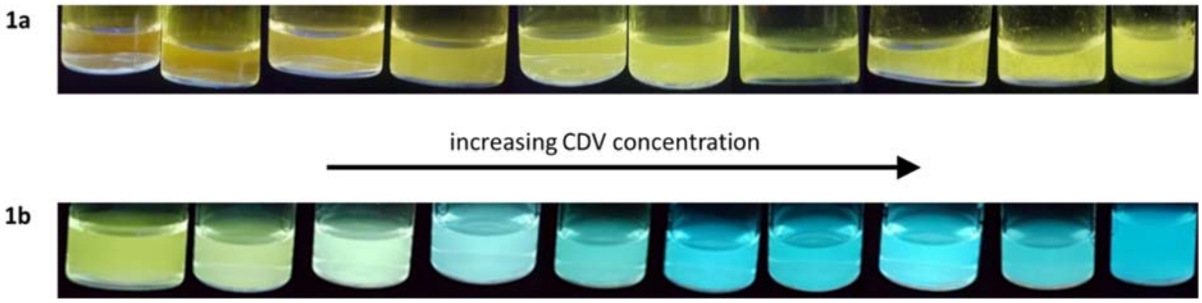

Figure S9: Pictures of 10 μM **1a**/**1b** with different concentrations of CDV (0-80 μM) under UV irradiation.

Cell culture experiments:

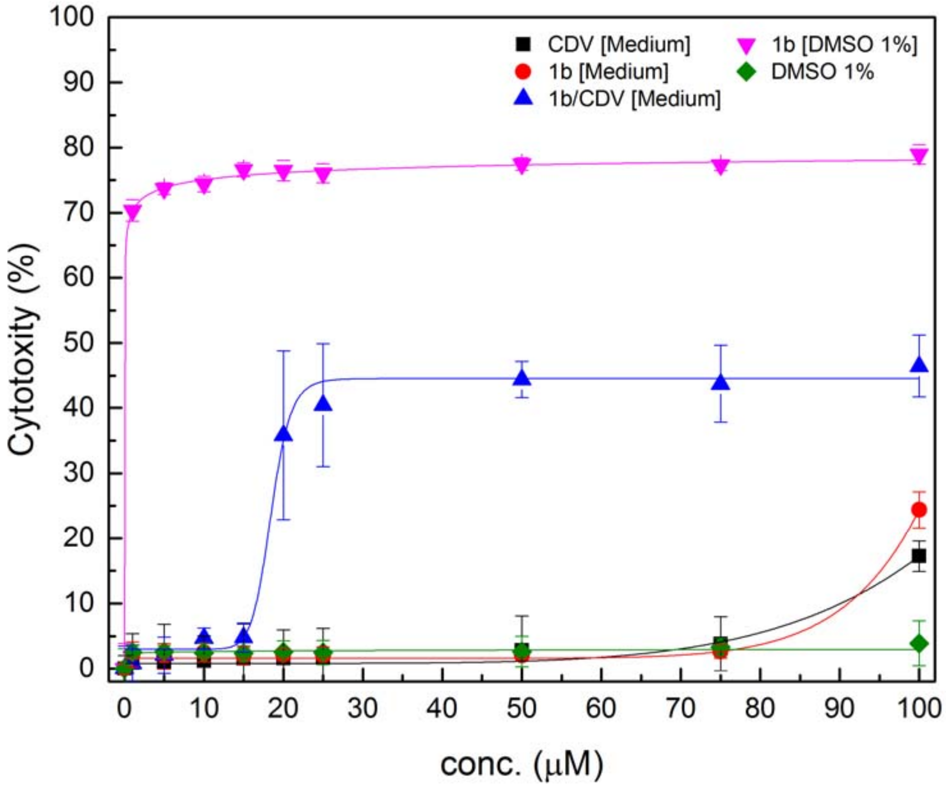

Figure S10: Cytotoxicity effect in % versus the final concentration ranging from 0 to 100 µM of CDV (black), **1b** (red) and **1b**@CDV (blue) prepared in assay medium or **1b** dissolved in DMSO (purple) and DMSO control (green) alone. HUVECs were treated with the respective substances for 2 h and LDH activity in the cell culture supernatant was measured as an indicator of cell lysis. Mean and standard deviations for each concentration substances were obtained from six (n = 6) independent experiments. The response curves were fitted to a growth sigmoidal function employing Origin 9.1 with a coefficient of determination R2 value [HUVECs for β-CDV (R2 = 0.98987), **1b** (R2 = 0.92848), **1b**@CDV (R2 = 0.99047, **1b** (DMSO) (R2 = 0.99573)].

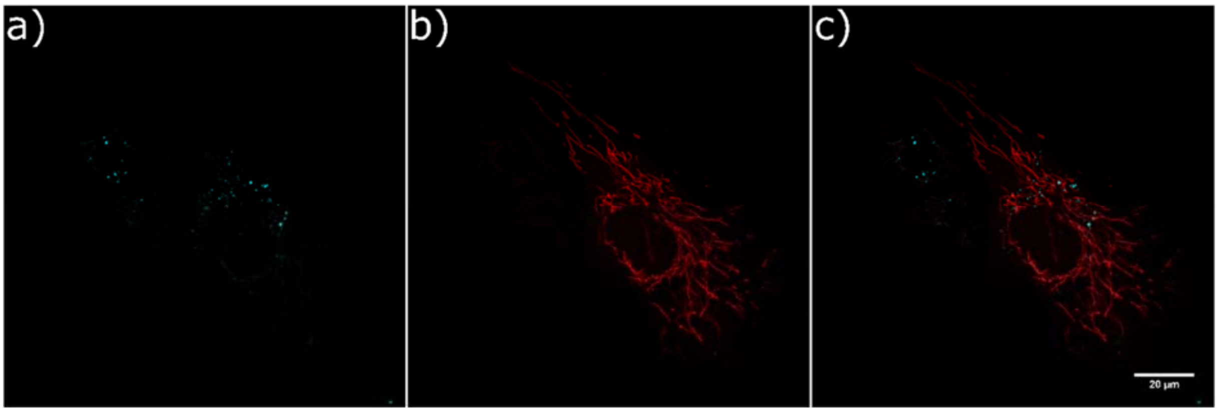

Figure S11: Live cell confocal microscopy pictures showing Mito-RFP transfected HUVECs 24 h after transfection followed by a 90 minute incubation time with **3**/CDV diluted in medium to final concentrations of 1.25 µM of **3** and 3.75 µM of CDV. **a)** Signal of **3** in blue (λ_{exc} = 405 nm, λ_{em} = 410-556 nm).; **b)** Mito-RFP channel showing mitochondria specific label; **c)** merge.

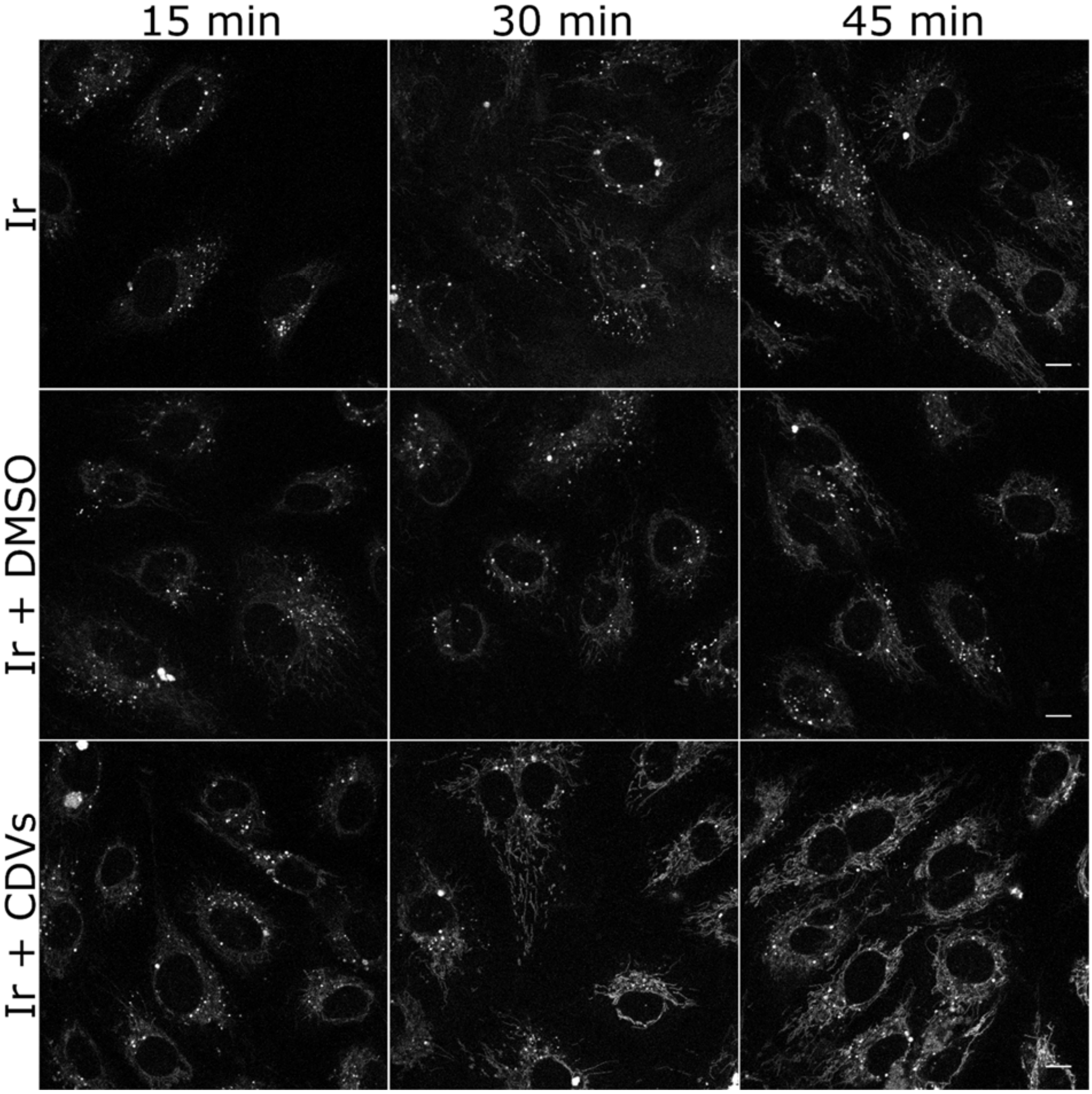

Figure S12: Live cell confocal microscopy pictures (λ_{exc} = 405 nm, λ_{em} = 410-556 nm) showing HUVECs after incubation with 1.25 μM **1b**, 1.25 μM **1b** and 1 % DMSO or 1.25 μM **1b** and 3.75 μM of CDV for 15, 30 or 45 min. Scale bars: 10 μm.

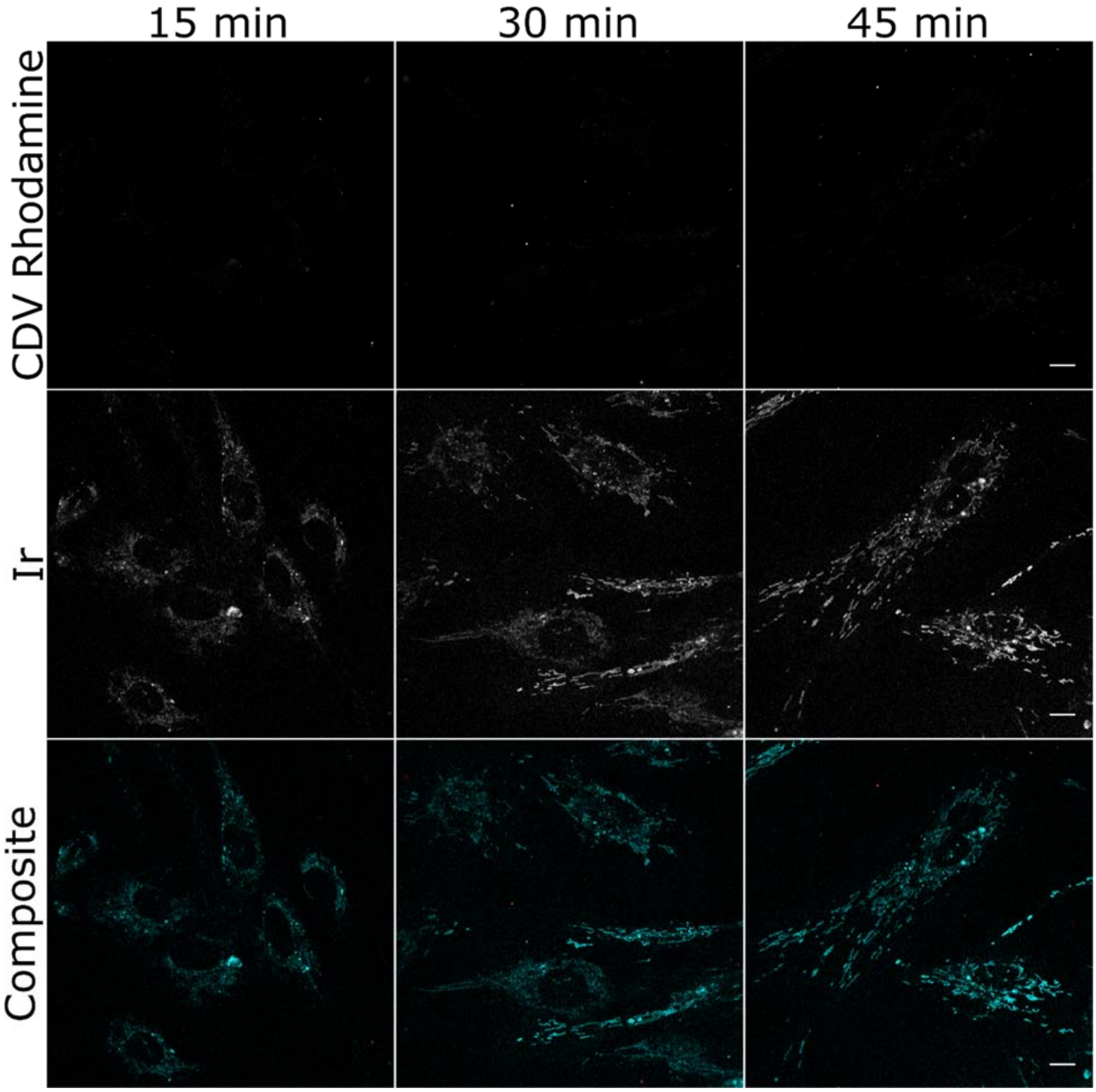

Figure S13: Live cell confocal microscopy pictures of HUVECs after incubation with 1.25 µM **1b** and 3.75 µM of CDV conjugated to rhodamine for 15, 30 or 45 min. Shown are the CDV rhodamine (upper row), the Ir at λ_{exc} = 405 nm (middle) and a merge (composite). Scale bars: 10 µm

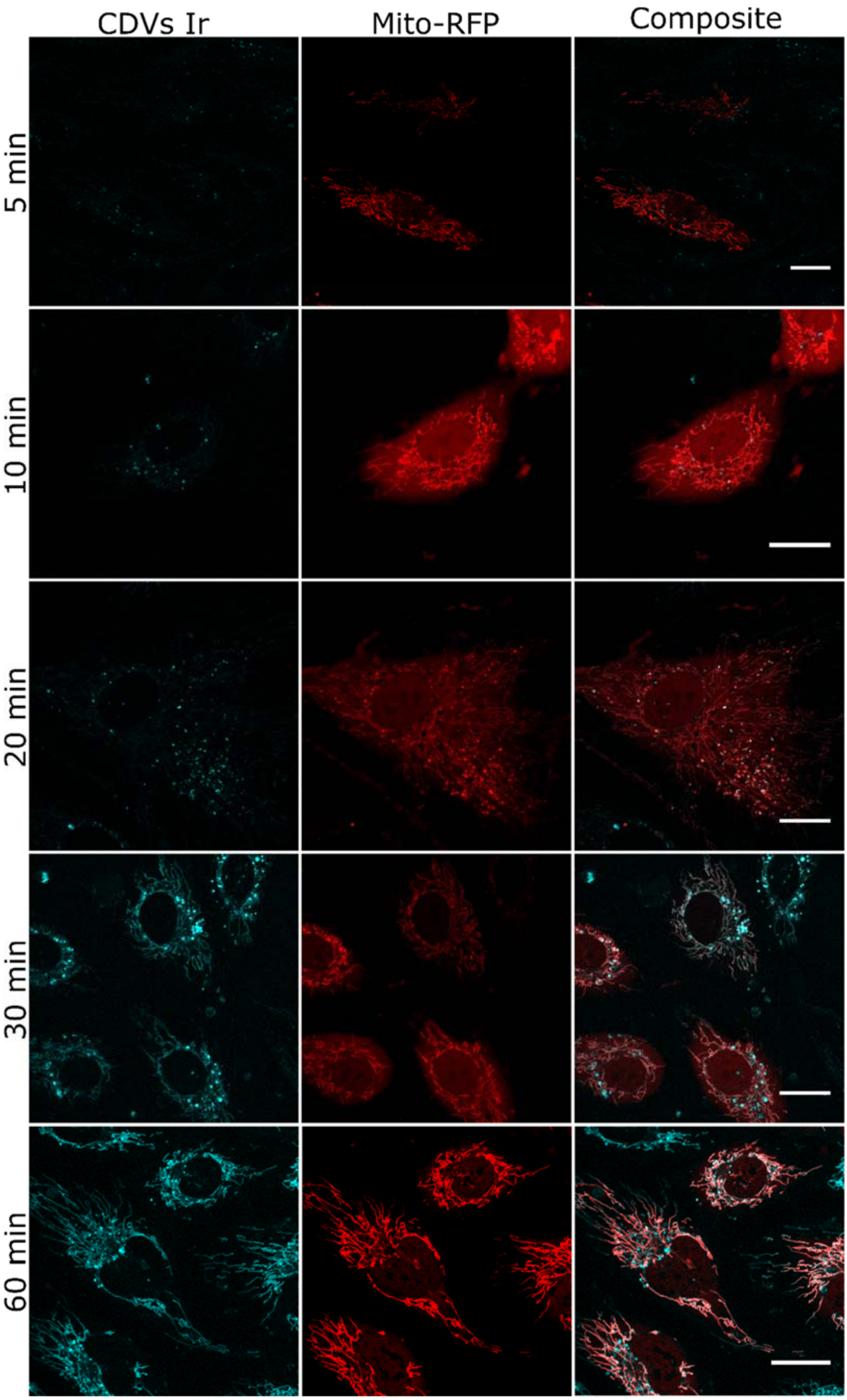

Figure S14: Live cell confocal microscopy pictures 24 h past transfection with Mito-RFP followed by incubation with **1b**-decorated CDV diluted in medium for 5, 10, 20, 30 or 60 min. The complex is shown in blue. Scale bar 20 µm.

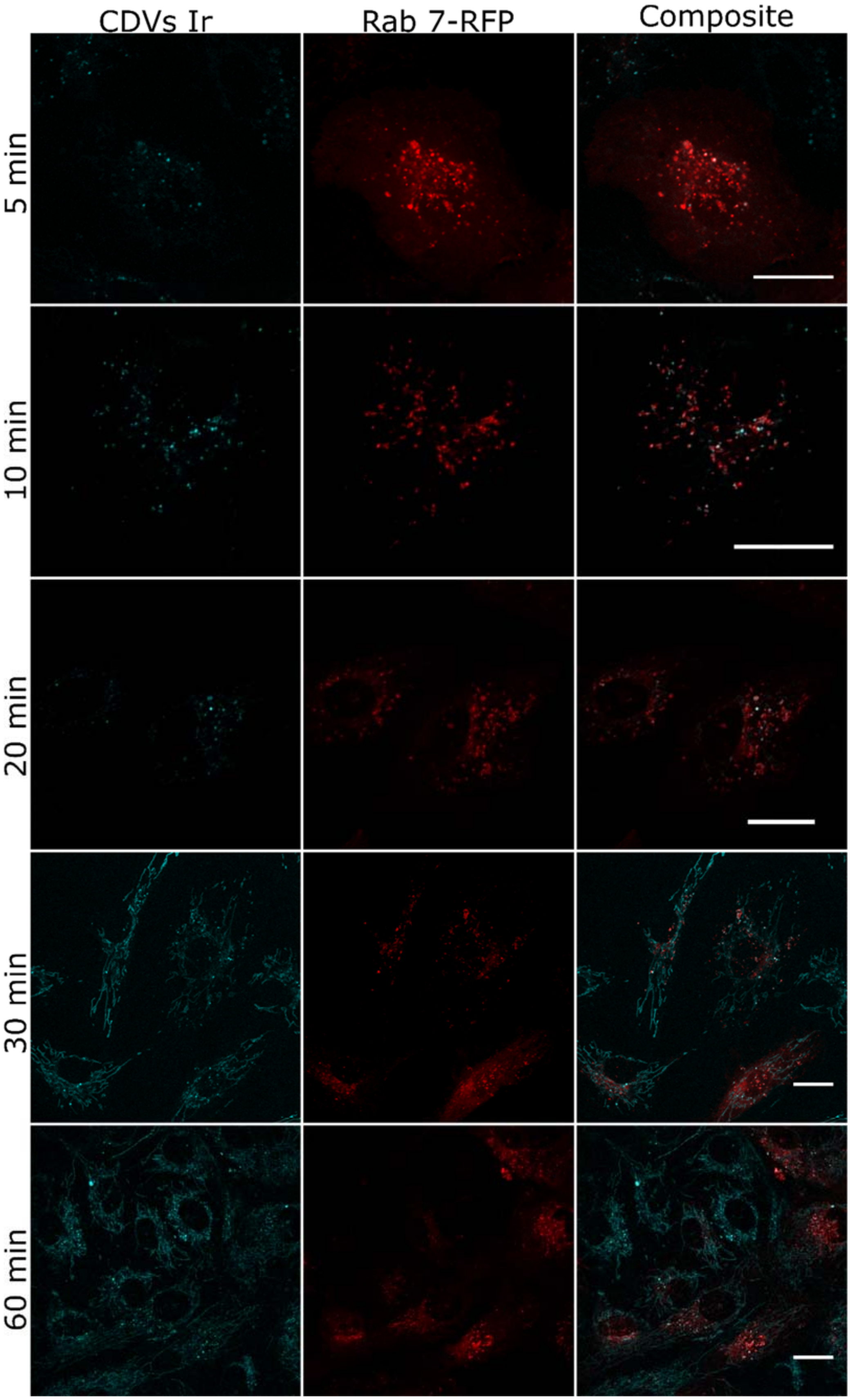

Figure S15: Live cell confocal microscopy pictures 24 h past transfection with Rab7-RFP followed by incubation with **1b**-decorated CDV diluted in medium for 5, 10, 20, 30 or 60 min. The complex is shown in blue. Scale bar 20 µm.

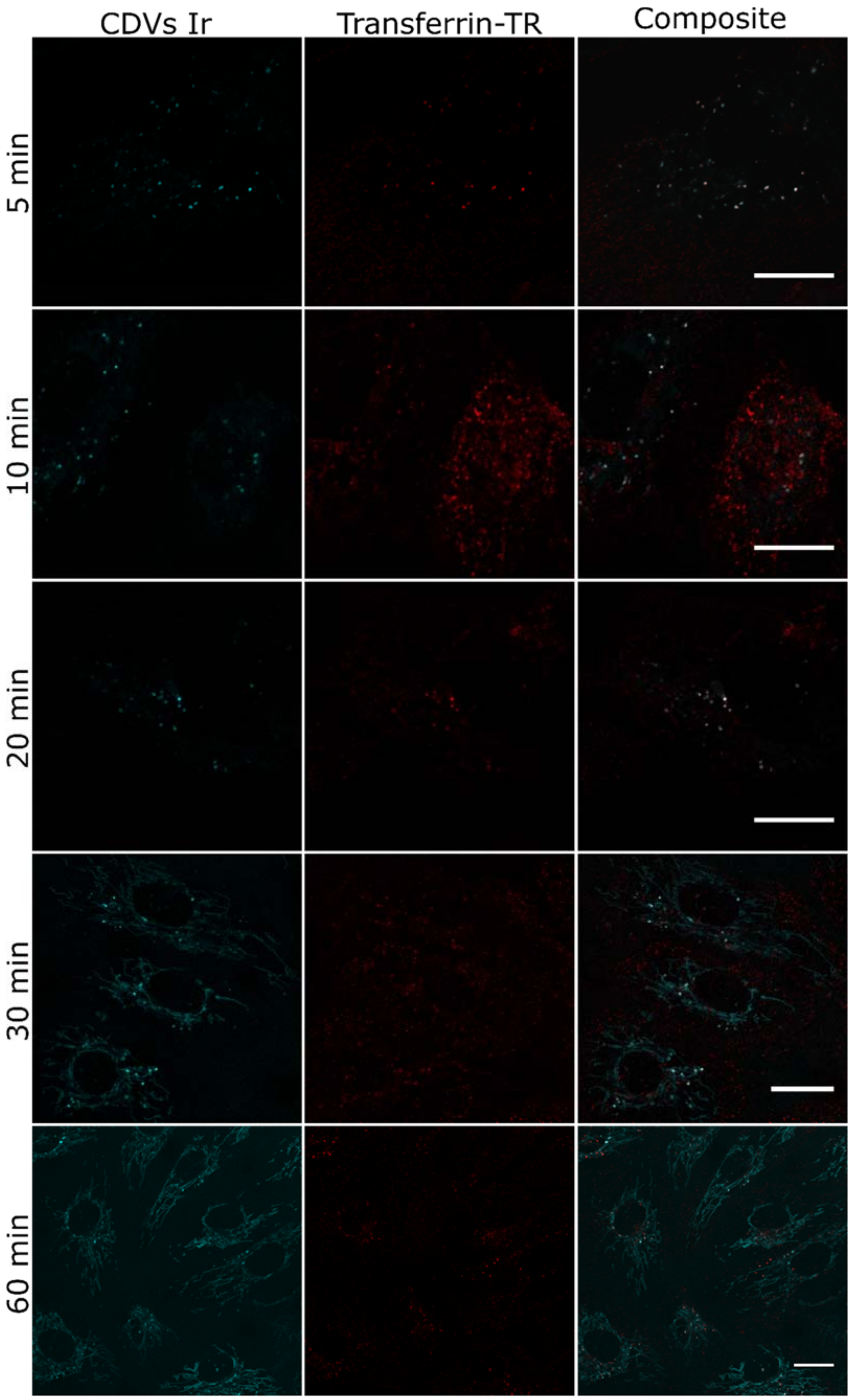

Figure S16: Live cell confocal microscopy pictures after 5 min treatment with 50 µg/ml transferrin-TexasRed followed by incubation with **1b**-decorated CDV diluted in medium for 5, 10, 20, 30 or 60 min. The complex is shown in blue. Scale bar 20 µm.

Synthesis

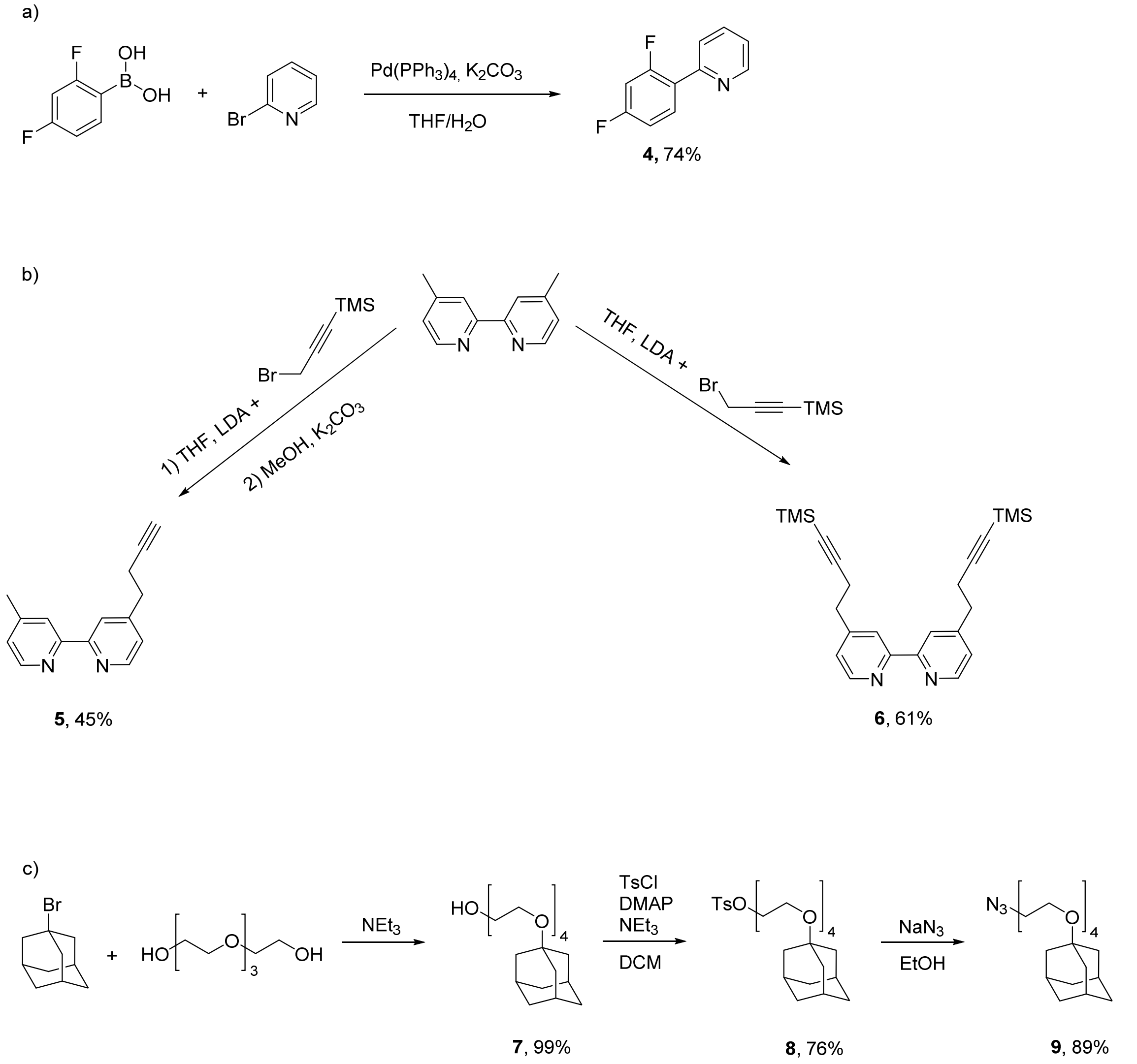

Figure S17: Synthesis of precursor **4**, **5**, **6** and **9**.

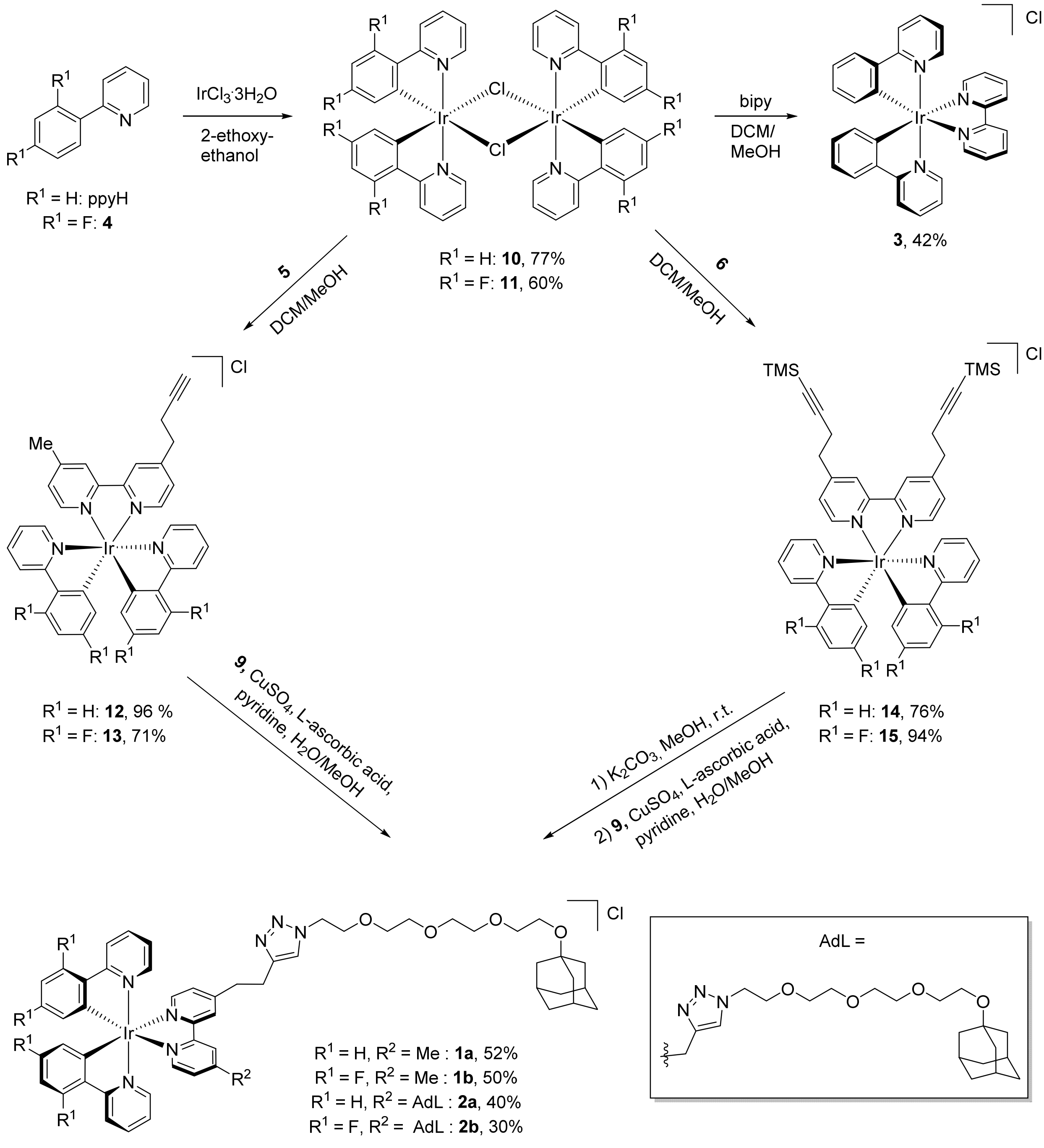

Figure S18: Synthesis of Ir complexes **1a**, **1b**, **2a**, **2b** and **3**.

2-(2,4-difluorophenyl)pyridine (4)

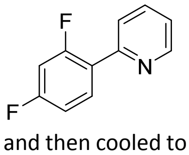

(2,4-difluorophenyl)boronic acid (2.17 g, 13.5 mmol, 1.08 equiv.), 2-bromopyridine (1.19 mL, 12.5 mmol, 1.0 equiv.) and potassium carbonate (4.32 g, 31.25 mmol, 4.3 equiv.) were added to a Schlenk flask containing THF:H_2O=25mL:5mL (v/v). The solution was bubbled by nitrogen for 10 minutes before $Pd(PPh_3)_4$ (0.40 g, 0.35 mmol, 0.03 equiv.) was added. The mixture was refluxed under nitrogen overnight and then cooled to room temperature and extracted by DCM (3 x 10 mL). The organic fractions were combined, washed with brine and dried over sodium sulfate. Filtration and evaporation under reduced pressure gave the crude product. The crude product was purified by column chromatography (DCM, Rf: 0.42). **4** was found as yellow liquid (1.77 g, 9.3 mmol, 74%).

^{1}H NMR (500 MHz, $CDCl_3$) δ (ppm): 8.71 (m, 1H), 8.00 (td, J = 8.8, 6.6 Hz, 1H), 7.79 – 7.71 (m, 2H), 7.30 – 7.21 (m, 1H), 7.01 (m, 1H), 6.92 (m, 1H).

The obtained results are in agreement with literature data.[9]

4-(But-3-yn-1-yl)-4`-methyl-2,2`-bipyridine (5)

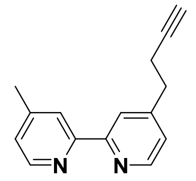

All flasks have to be dried and this reaction was conducted in nitrogen. 4,4'-dimethyl-2,2'-bipyridine (465 mg, 2.52 mmol, 1.0 equiv.) was dissolved in dry THF (25 ml) and cooled to -78 °C for 15 minutes (dry ice and acetone bath). Another flask containing 5 mL THF and distilled diisopropylamine (0.43 ml, 3.10 mmol, 1.2 equiv.) was cooled to -78 °C, and *n*-butyllithium (1.59 ml, 2.5 M, 2.54 mmol, 1.0 equiv.) was added dropwise stirring for 15 minutes to prepare the lithium diisopropylamide (LDA). The freshly prepared LDA solution was added dropwise via a cannula to the THF solution of 4,4'-dimethyl-2,2-bipyridine, and the solution was stirred for 1h. The (3-bromoprop-1-yn-1-yl)trimethylsilane (0.5 mL, 3.53 mmol, 1.4 equiv.) was dissolved in THF (3 mL), cooled to -78°C and added dropwise via a cannula to the 4,4'-dimethyl-2,2-bipyridine solution. The solution was stirred at this temperature for 0.5 h before being allowed to warm up to room temperature, and then stirred overnight. The mixture was poured into distilled water and extracted three times with DCM. The organic layer was collected and dried with Na_2SO_4. Filtration and evaporation under reduced pressure gave the crude product. The crude product was purified by column chromatography (silica, Hexane/ethyl acetate= 3:1, Rf: 0.38) to give a colourless solid which was then redissolved in methanol (20 mL) with K_2CO_3 (600 mg, 4.34 mmol, 1.7 equiv.) and stirred for 4 hours at room temperature. DCM and water were used for three times extraction after removing the methanol. The organic fractions were combined, washed with a portion of brine and dried over Na_2SO_4. Filtration and evaporation under reduced pressure gave the crude product. This solid was kept in vacuum for 1 h to remove remaining TMS and obtain the N^N ligand **5** as white solid. (160 mg, 0.72 mmol, 28 %).

^{1}H NMR (300 MHz, $CDCl_3$) δ(ppm): 8.59 (dd, *J* = 17.3, 5.0 Hz, 2H, Ar-H), 8.27 (d, *J* = 9.7 Hz, 2H, Ar-H), 7.20 (dd, *J* = 21.2, 5.0 Hz, 2H, Ar-H), 2.96 (t, *J* = 7.4 Hz, 2H, CH_2), 2.61 (td, *J* = 7.4, 2.6 Hz, 2H, CH_2), 2.47 (s, 3H, CH_3), 2.02 (t, *J* = 2.6 Hz, 1H, C≡CH).

^{13}C NMR (126 MHz, CD_2Cl_2) δ (ppm): 156.21, 155.77, 150.10, 149.01, 148.83, 148.11, 124.67, 123.82, 121.72, 120.90, 82.99, 69.16, 34.13, 20.97, 19.31.

HRMS (NSI^+): m/z calculated: 223.1230 $[(C_{15}H_{14}N_2)H]^+$, measured: 223.1230.

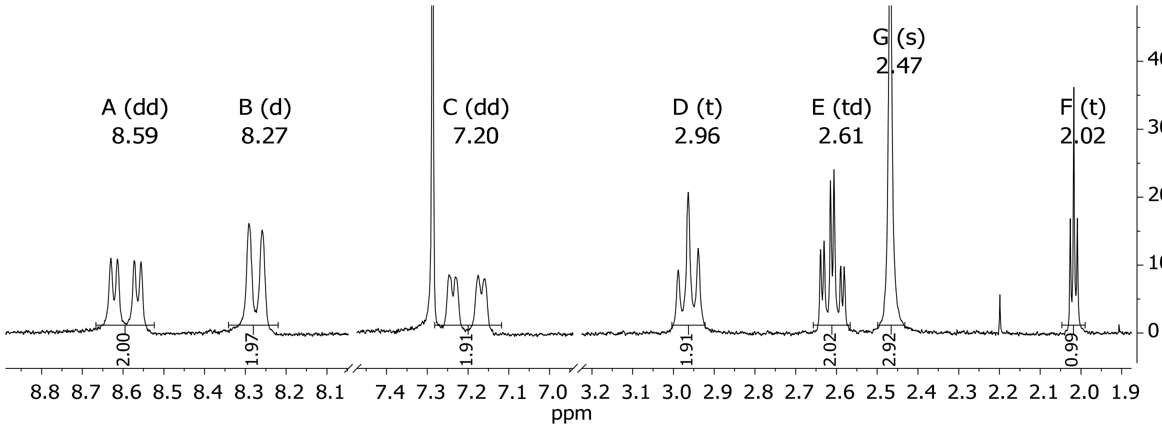

Figure S19: ^{1}H-NMR of 4-(But-3-yn-1-yl)-4`-methyl-2,2`-bipyridine (**5**).

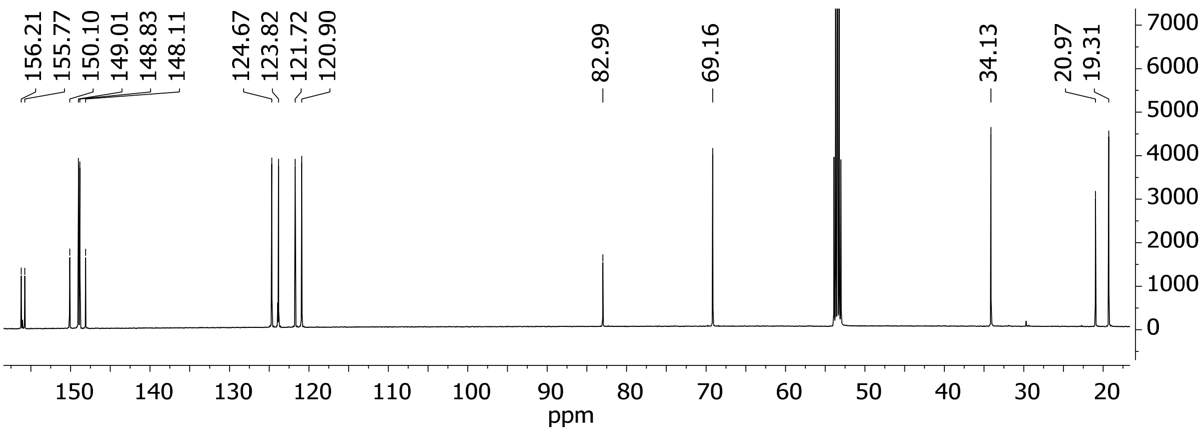

Figure S20: ^{13}C-NMR of 4-(But-3-yn-1-yl)-4`-methyl-2,2`-bipyridine (**5**).

4-(4-(Trimethylsilyl)but-3-yn-1-yl)-4`-methyl-2,2`-bipyridine (6)

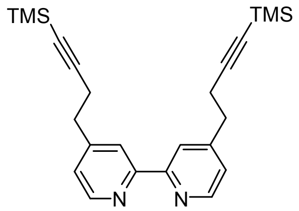

Lithium diisopropylamide (LDA) was prepared by adding *n*-butyllithium (3.44 ml, 1.6 M, 5.50 mmol, 2.4 equiv.) dropwise to distilled diisopropylamine (1.16 ml, 8.36 mmol, 3.6 equiv.) in THF (5 mL) at -78°C. 4-4′-dimethyl-2,2`-bipyridine (0.43 g, 2.31 mmol, 1.0 equiv.) in dry THF (25 mL) was added dropwise to the freshly prepared LDA at -78 °C under nitrogen atmosphere. The resulting solution was stirred in these conditions for one hour. After that, this solution was added through a cannula to a solution of 3-bromo-1-(trimethylsilyl)-1-propyne (0.92 mL, 6.47 mmol, 2.8 equiv.) in dry THF (3 mL). The resulting solution was stirred in these conditions overnight allowing it to reach room temperature. The mixture was poured into distilled water, extracted with DCM and dried over $NaSO_4$. After evaporation of the solvent, the residue was purified by column chromatography (hexane/ethyl acetate (20/1), Rf: 0.57) yielding compound **7** as a white solid (61 %).

^{1}H NMR (500 MHz, $CDCl_3$) δ (ppm): 8.60 (d, *J* = 5.6 Hz, 2H), 8.31 (s, 2H), 7.22 (dd, *J* = 5.0, 1.7 Hz, 2H), 2.93 (t, *J* = 7.3 Hz, 4H), 2.61 (t, *J* = 7.3 Hz, 4H), 0.14 (s, 18H).

^{13}C NMR (126 MHz, $CDCl_3$) δ (ppm): 156.12, 150.33, 149.00, 124.10, 121.37, 105.50, 86.15, 34.39, 21.08, 0.01.

HRMS (ASAP): m/z calculated: 405.2182 [$(C_{24}H_{32}N_2Si_2)H]^+$, measured: 405.2183.

Anal. Calcd. for $C_{24}H_{32}N_2Si_2$: C, 71.23; H, 7.97; N, 6.92. measured: C, 69.98; H, 7.76; N, 6.91.

The obtained results are in agreement with literature data.[10]

2-(2-(2-(2-((adamant-1-yl)oxy)ethoxy)ethoxy)ethoxy)ethanol (7)

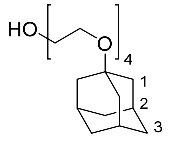

1-bromoadamantane (5.00 g, 23.2 mmol, 1.0 equiv.), triethylamine (9.6 mL, 69.6 mmol, 3.0 equiv.) and tetraethylene glycol (100 mL) were added to a round bottom flask and stirred overnight at 180°C. After cooling to RT, DCM (150 mL) was added and the mixture was washed with 2 M HCl_{aq} (4×50 mL) and brine (50 mL). The organic layer was dried over $MgSO_4$. Finally, the solvent was removed and product **7** was recovered as brown oil (7.62 g, 23.2 mmol, 99%).

^{1}H-NMR (300 MHz, $CDCl_3$) δ (ppm): 3.73 – 3.68 (m, 2H, CH_2O), 3.65 (d, *J* = 2.7 Hz, 8H, CH_2O), 3.61 – 3.54 (m, 6H, CH_2O), 2.97 (s, 1H, OH), 2.12 (s, 3H, CH-2), 1.72 (d, *J* = 3.0 Hz, 6H, CH_2-1), 1.66 – 1.52 (m, 6H, CH_2-3).

^{13}C-NMR (75 MHz, $CDCl_3$) δ (ppm): 72.67, 72.43, 71.37, 70.72, 70.69, 70.65, 70.43, 61.82, 59.35, 41.52 (CH_2, C-1), 36.54 (CH_2, C-3), 30.59 (CH, C-2).

HRMS (ESI): m/z calculated: 351.2142 [$(C_{18}H_{32}O_5)Na]^+$, measured: 351.2138.

The obtained results are in agreement with literature data.[11]

2-(2-(2-(2-((adamant-1-yl)oxy)ethoxy)ethoxy)ethoxy)ethyl 4-methyl benzenesulfonate (8)

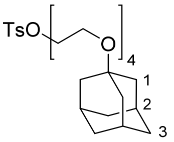

2-(2-(2-(2-((adamant-1-yl)oxy)ethoxy)ethoxy)ethoxy)ethanol (**7**) (7.62 g, 23.2 mmol, 1.0 equiv.) and 4-DMAP (0.15 g, 1.2 mmol, catalytic) were dissolved in DCM (250 mL). First triethylamine (4.85 mL, 35.0 mmol, 1.5 equiv.), then tosylchloride (4.86 g, 25.5 mmol, 1.1 equiv.) dissolved in DCM (150 mL) were added dropwise at 0°C. The mixture was stirred overnight at RT, washed with H_2O (2×200 mL) and 2 M HCl_{aq} (200 mL) and dried over $MgSO_4$. After evaporating the solvent and purification by column chromatography (EtOAc, Rf: 0.62), product **8** was recovered as yellow oil (8.54 g, 17.7 mmol, 76%).

^{1}H-NMR (400 MHz, $CDCl_3$) δ (ppm): 7.78 (d, *J* = 8.3 Hz, 2H, Ar-H), 7.33 (d, *J* = 8.1 Hz, 2H, Ar-H), 4.17 – 4.12 (m, 2H, CH_2OTs), 3.70 – 3.65 (m, 2H, CH_2O), 3.65 – 3.49 (m, 12H, CH_2O), 2.43 (s, 3H, $ArCH_3$), 2.12 (s, 3H, CH-2), 1.72 (d, *J* = 3.0 Hz, 6H, CH_2-1), 1.59 (q, *J* = 12.1 Hz, 6H, CH_2-3).

^{13}C-NMR (75 MHz, $CDCl_3$) δ (ppm): 144.87 (Cq, C-S), 133.08 (Cq, C-CH_3), 129.91 (CH, CAr), 128.08 (CH, CAr), 72.34, 71.37, 70.84, 70.73, 70.67, 70.62, 69.35, 68.76, 59.34, 41.56 (CH_2, C-1), 36.55 (CH_2, C-3), 30.59 (CH, C-2), 21.76 (CH_3, Ar-CH3).

HRMS (ESI): m/z calculated: 505.2230 $[(C_{25}H_{38}O_7S)Na]^+$, measured: 505.2229.

The obtained results are in agreement with literature data.[12]

2-(2-(2-(2-((adamant-1-yl)oxy)ethoxy)ethoxy)ethoxy)ethyl azide (9)

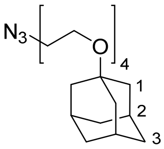

8 (3.0 g, 6.3 mmol, 1.0 equiv.) and NaN_3 (813 mg, 12.5 mmol, 2.0 equiv.) were dissolved in EtOH (50 mL) and stirred at 85°C for 16 h. After cooling down to room temperature the solvent was removed, and the residue was dissolved in DCM. The organic phase was washed with water and dried over $MgSO_4$. Purification by column chromatography (EtOAc) gave product **9** as a colorless oil (1.96 g, 5.5 mol, 89 %).

^{1}H-NMR (400 MHz, $CDCl_3$) δ (ppm): 3.72 – 3.63 (m, 10H, 5xCH_2), 3.62 – 3.54 (m, 4H, 2xCH_2), 3.38 (t, *J* = 5.1 Hz, 2H, CH_2), 2.13 (s, 3H, CH-2), 1.73 (d, *J* = 2.9 Hz, 6H, CH_2-1), 1.66 – 1.53 (m, 6H, CH_2-3).

^{13}C-NMR (101 MHz, $CDCl_3$) δ (ppm): 72.34, 71.40, 70.85, 70.83, 70.78, 70.75, 70.16, 59.38, 50.82, 41.61 (CH_2, C-1), 36.59 (CH_2, C-3), 30.63 (CH, C-2).

HRMS (ESI): m/z calculated: 376.2212 $[(C_{18}H_{31}N_3O_4)Na]^+$, measured: 376.2213.

The obtained results are in agreement with literature data.[13]

General procedure 1: Synthesis of $[Ir(C^\wedge N)_2]_2Cl_2$ dimers

Ir dimers were synthesized following the procedure of Nanoyama[14]. The corresponding ligand (2.2 equiv.) and $IrCl_3 \cdot 3H_2O$ (1.0 equiv.) were dissolved in 2-ethoxyethanol to give a concentration of 0.02 M. The mixture was degassed by multiple vacuum and N_2 purging cycles. Afterwards, it was refluxed at 140°C for overnight, cooled to room temperature and the precipitate was filtered and washed with water, hexane and diethyl ether. The product was used without any further purification.

$[Ir(ppy)_2Cl]_2$ (10)

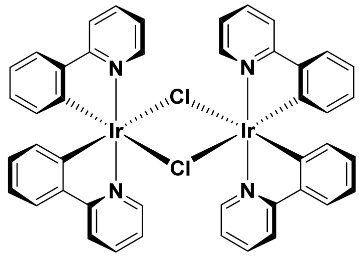

2-phenylpyridine (97 mg, 0.66 mmol, 2.2 equiv.) and $IrCl_3 \cdot 3H_2O$ (100 mg, 0.28 mmol, 1.0 equiv.) were added to a Schlenk tube containing 2-ethoxyethanol (15 mL). Product **10** was obtained as yellow powder (231 mg, 0.22 mmol, 77 %).

^{1}H NMR (300 MHz, CD_2Cl_2) δ (ppm): 9.27 – 9.22 (m, 4H), 7.96 – 7.91 (m, 4H), 7.83 – 7.76 (m, 4H), 7.58 – 7.53 (m, 4H), 6.86 – 6.78 (m, 8H), 6.64 – 6.57 (m, 4H), 5.89 – 5.84 (m, 4H).

The obtained results are in agreement with literature data.[15]

$[Ir(dfppy)_2Cl]_2$ (11)

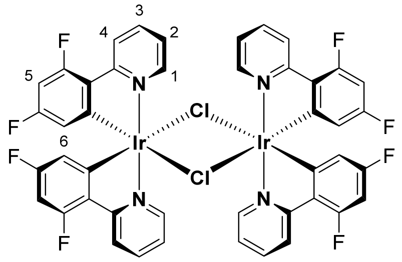

4 (400 mg, 2.08 mmol, 2.2 equiv.) and $IrCl_3 \cdot 3H_2O$ (334 mg, 0.95 mmol, 1.0 equiv.) were added to a Schlenk tube containing 2-ethoxyethanol (45 mL). Product **11** was obtained as yellow powder (344 mg, 0.28 mmol, 60 %).

^{1}H NMR (500 MHz, CD_2Cl_2) δ (ppm): 9.12 (d, J = 5.7 Hz, 4H, CH-1), 8.33 (d, J = 8.4 Hz, 4H, CH-4), 7.87 (t, J = 7.9 Hz, 4H, CH-3), 6.87 (t, J = 6.7 Hz, 4H, CH-2), 6.46 – 6.28 (m, 4H, CF-CH), 5.31 – 5.26 (m, 4H, CF-CH).

The obtained results are in agreement with literature data.[16]

General procedure 2: Synthesis of [Ir(C^N)$_2$(N^N)]Cl complexes

[Ir(C^N)$_2$]$_2$Cl$_2$ dimer and the ancillary N^N ligand (2.5 equiv.) were added to a Schleck tube containing a mixture of DCM and MeOH. The mixture was degassed by multiple vacuum and N_2 purging cycles. Afterwards, it was stirred at 55 °C for 19 h under nitrogen atmosphere and cooled to room temperature. The solvent was evaporated under vacuum and the product was purified by flash column chromatography.

Ir[(2-ppy)$_2$(2,2'-bipy)]Cl complex (3)

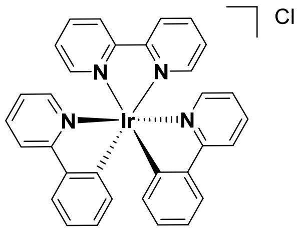

3 was synthesized following **general procedure 2**. Compound **10** (50 mg, 0.05 mmol, 1.0 equiv.) and 2,2'-bipyridine (18 mg, 0.12 mmol, 2.5 equiv.) were added to a Schleck tube containing a mixture of DCM and MeOH (2:1, 15 mL). **3** was obtained after column chromatography (DCM/MeOH gradient 20:1 to 10:1; Rf: 0.53 (MeOH/DCM = 1:19 on silica)) as yellow solid (29 mg, 0.042 mmol, 42 %).

^{1}H NMR (400 MHz, CD$_2$Cl$_2$) δ (ppm): 9.14 (d, *J* = 8.2 Hz, 2H), 8.23 (dd, *J* = 8.1, 1.6 Hz, 2H), 8.08 – 7.97 (m, 4H), 7.90 – 7.75 (m, 4H), 7.59 – 7.47 (m, 4H), 7.11 (td, *J* = 7.6, 1.2 Hz, 2H), 7.08 – 6.92 (m, 4H), 6.44 – 6.32 (m, 2H)

The obtained results are in agreement with literature data.[17]

[Ir(ppy)$_2$[4-(but-3-yn-1-yl)-4`-methyl-2,2`-bipyridine]]Cl complex (12)

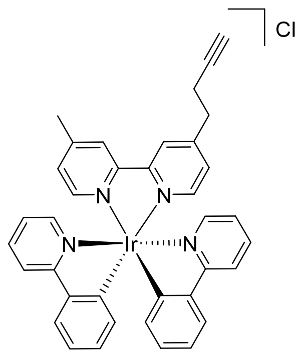

12 was synthesized following **general procedure 2**. **10** (50 mg, 0.05 mmol, 1.0 equiv.) and **5** (26 mg, 0.12 mmol, 2.5 equiv.) were added to a Schleck tube containing a mixture of DCM and MeOH (2:1, 15 mL). Ir complex **12** was obtained after column chromatography (DCM/MeOH gradient 20:1 to 10:1) as yellow solid (36 mg, 0.048 mmol, 96 %).

^{1}H NMR (300 MHz, CD$_2$Cl$_2$) δ (ppm): 8.34 (d, *J* = 15.4 Hz, 2H), 7.98 (d, *J* = 8.3 Hz, 2H), 7.92 (d, *J* = 5.7 Hz, 1H), 7.88 – 7.71 (m, 5H), 7.52 (dd, *J* = 11.6, 5.8 Hz, 2H), 7.37 (d, *J* = 5.6 Hz, 1H), 7.27 (d, *J* = 5.8 Hz, 1H), 7.12 – 7.05 (m, 2H), 7.05 – 6.98 (m, 2H), 6.95 (td, *J* = 7.4, 1.3 Hz, 2H), 6.33 (dd, *J* = 7.6, 3.9 Hz, 2H), 3.07 (t, *J* = 6.9 Hz, 2H), 2.66 (td, *J* = 6.9, 2.7 Hz, 2H), 2.61 (s, 3H), 2.08 (t, *J* = 2.6 Hz, 1H).

^{13}C NMR (126 MHz, CD$_2$Cl$_2$) δ (ppm): 167.82/167.80, 156.06, 155.75, 154.03, 152.39, 150.73, 149.65, 149.42, 148.57/148.50, 143.77/143.74, 137.95, 131.68/131.63, 130.53, 128.71, 128.14, 126.92, 126.28, 124.78, 123.16, 122.41, 119.69/119.67, 82.73, 69.77, 33.74, 21.17, 18.86.

FTMS (NSI): m/z calculated [$C_{37}H_{30}N_4Ir$]$^+$: 723.12; measured: 723.21.

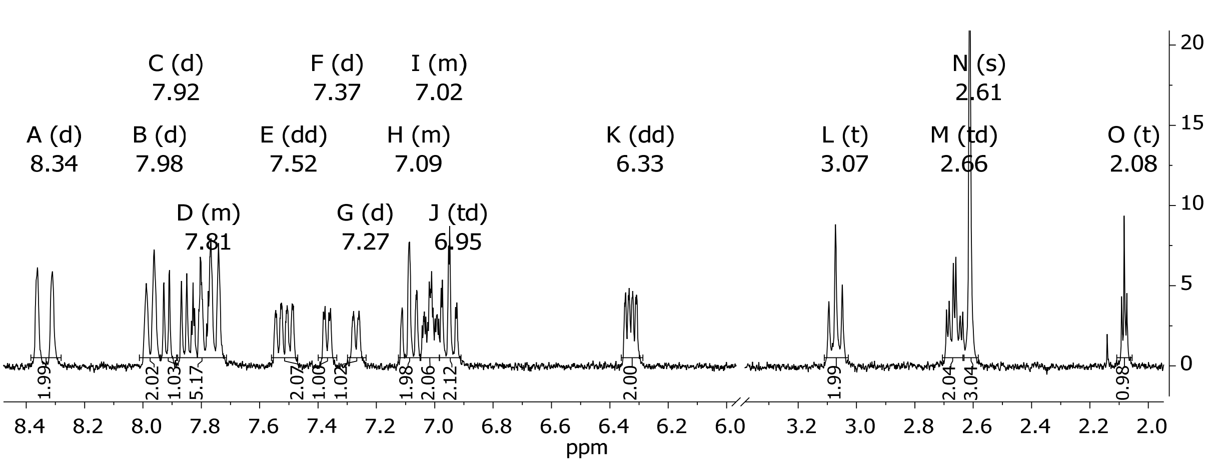

Figure S21: ^{1}H-NMR of [Ir(ppy)$_2$[4-(but-3-yn-1-yl)-4`-methyl-2,2`-bipyridine]]Cl (**12**).

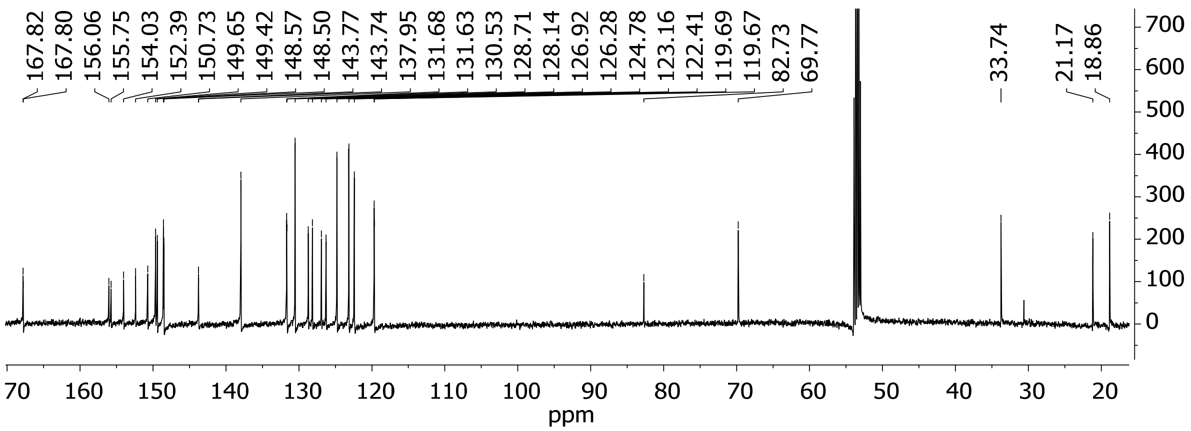

Figure S22: ^{13}C-NMR of [Ir(ppy)$_2$[4-(but-3-yn-1-yl)-4`-methyl-2,2`-bipyridine]]Cl (**12**).

[Ir(dfppy)$_2$[4-(but-3-yn-1-yl)-4`-methyl-2,2`-bipyridine]]Cl complex (13)

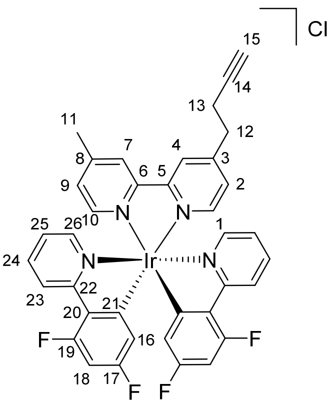

13 was synthesized following **general procedure 2**. **11** (340 mg, 0.28 mmol, 1.0 equiv.) and **5** (156 mg, 0.7 mmol, 2.5 equiv.) were added to a Schleck tube containing a mixture of DCM and MeOH (1:1, 10 mL). Ir complex **13** was obtained after column chromatography (DCM/MeOH gradient 100:0 to 60:40) as yellow solid (366 mg, 0.44 mmol, 71 %).

^{1}H NMR (500 MHz, CD_2Cl_2) δ (ppm): 9.65 (s, 1H, ArH-4), 9.61 (s, 1H, ArH-7), 8.31 (dt, *J* = 8.6, 1.5 Hz, 2H, ArH-23), 7.85 – 7.79 (m, 3H, ArH-24, ArH-1), 7.75 (d, *J* = 5.6 Hz, 1H, ArH-10), 7.52 (dd, *J* = 5.9, 0.9 Hz, 1H, ArH-26), 7.48 (dd, *J* = 5.9, 0.9 Hz, 1H, ArH-26), 7.38 (dd, *J* = 5.7, 1.7 Hz, 1H, ArH-2), 7.26 (dd, *J* = 5.7, 0.9 Hz, 1H, ArH-9), 7.04 (dddd, *J* = 7.5, 5.9, 3.5, 1.4 Hz, 2H, ArH-25), 6.59 (dddd, *J* = 11.5, 9.1, 2.4, 1.2 Hz, 2H, ArH-18), 5.75 (ddd, *J* = 8.4, 7.2, 2.4 Hz, 2H, ArH-16), 3.16 (t, *J* = 7.0 Hz, 2H, CH_2-12), 2.86 – 2.78 (m, 2H, CH_2-13), 2.68 (s, 3H, CH_3-11), 2.01 (t, *J* = 2.6 Hz, 1H, CH-15).

^{13}C NMR (126 MHz, CD_2Cl_2) δ (ppm): 156.45, 156.13, 155.55, 155.07, 153.94 (C_q-3, C_q-5, C_q-6, C_q-8, C_q-22), 149.88 (CH-1), 149.65 (CH-10), 149.21 (CH-26a), 149.15 (CH-26b), 139.52 (CH-24), 129.53 (CH-9), 129.00 (CH-2), 128.31 (CH-7), 127.68 (CH-4), 124.36 (CH-23a), 124.20 (CH-23b), 124.06 (CH-25), 114.56, 114.51, 114.45, 114.38 (CH-16a/b, C_q-21a/b), 99.36 (CH-18a), 99.14 (CH-18b), 83.29 (C_q-14), 70.31 (CH-15), 34.29 (CH_2-12), 21.73 (CH_3-11), 19.38 (CH_2-13).

^{19}F {^{1} H} NMR (377 MHz, CD_2Cl_2) δ (ppm): -106.78 (d, *J* = 1.9 Hz), -106.81 (d, *J* = 1.9 Hz), -109.11 (d, *J* = 1.7 Hz), -109.14 (d, *J* = 1.8 Hz).

FTMS (NSI): m/z calculated: 795.1723 $[C_{37}H_{26}F_4N_4Ir]^+$, measured: 795.1702.

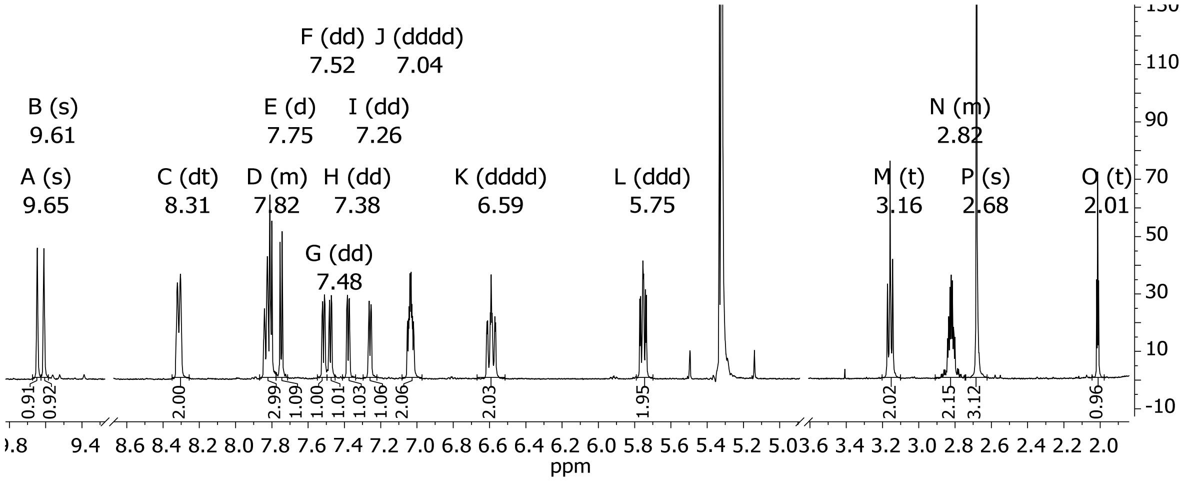

Figure S23: ^{1}H-NMR of [Ir(dfppy)$_2$[4-(but-3-yn-1-yl)-4`-methyl-2,2`-bipyridine]]Cl (**13**).

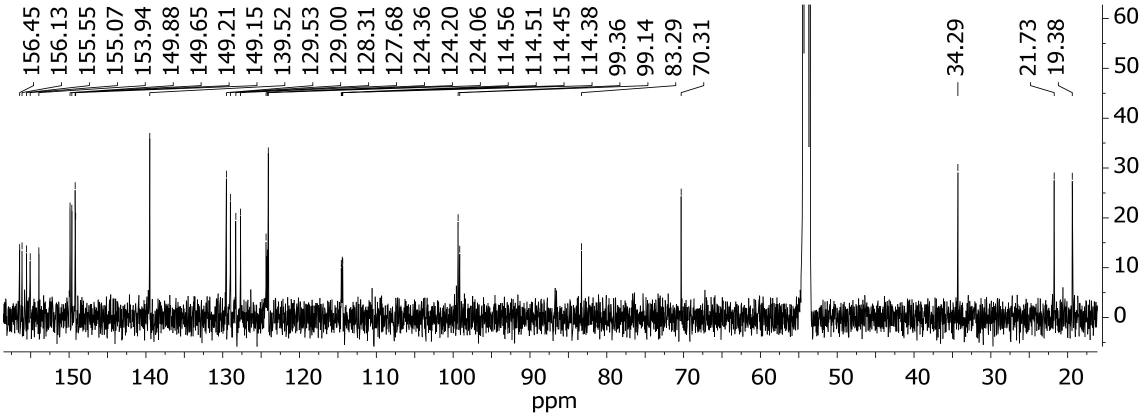

Figure S24: ^{13}C-NMR of [Ir(dfppy)$_2$[4-(but-3-yn-1-yl)-4`-methyl-2,2`-bipyridine]]Cl (**13**).

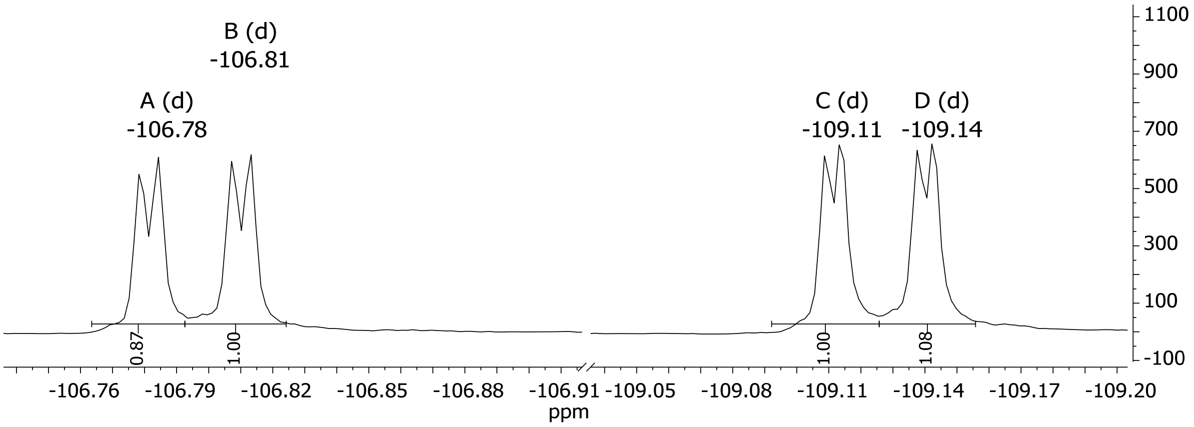

Figure S25: ^{19}F-NMR of [Ir(dfppy)$_2$[4-(but-3-yn-1-yl)-4`-methyl-2,2`-bipyridine]]Cl (**13**).

[Ir(ppy)$_2$-[4,4'-bis(4-(trimethylsilyl)but-3-yn-1-yl)-2,2'-bipyridine]]Cl (14)

14 was synthesized following **general procedure 2**. **10** (268 mg, 0.25 mmol, 1.0 equiv.) and **6** (253 mg, 0.625 mmol, 2.5 equiv.) were added to a Schleck tube containing a mixture of DCM and MeOH (2:1, 9 mL). Ir complex **14** was obtained after column chromatography (DCM/MeOH gradient 20:1 to 10:1) as yellow solid (76 %).

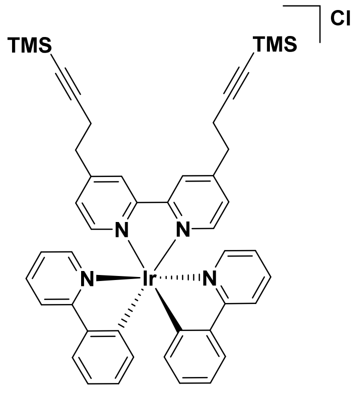

^{1}H NMR (500 MHz, CD_2Cl_2) δ (ppm): 9.50 (d, *J* = 7.7 Hz, 2H), 7.98 (d, *J* = 8.2 Hz, 2H), 7.85 (d, *J* = 5.6 Hz, 2H), 7.83 – 7.78 (m, 2H), 7.77 (d, *J* = 7.8 Hz, 2H), 7.56 (d, *J* = 6.4 Hz, 2H), 7.33 (d, *J* = 5.6 Hz, 2H), 7.09 (t, *J* = 7.5 Hz, 2H), 7.01 (t, *J* = 6.6 Hz, 2H), 6.96 (t, *J* = 8.0 Hz, 2H), 6.38 – 6.33 (m, 2H), 3.13 (dtd, *J* = 20.8, 13.8, 7.0 Hz, 4H), 2.83 (t, *J* = 7.0 Hz, 4H), 0.04 (s, 18H).

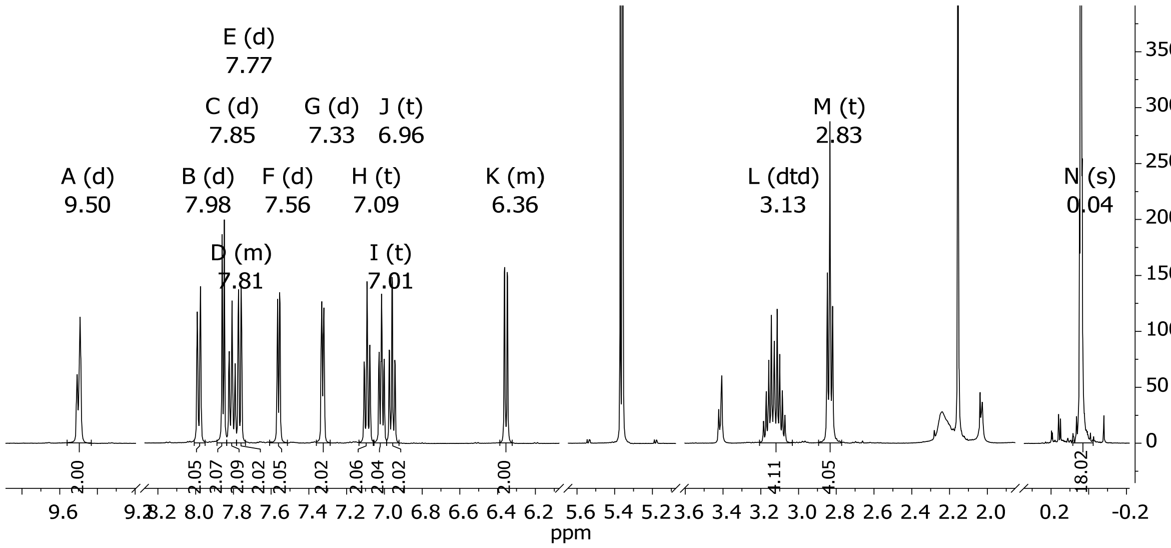

Figure S26: ^{1}H-NMR of [Ir(ppy)$_2$-[4,4'-bis(4-(trimethylsilyl)but-3-yn-1-yl)-2,2'-bipyridine]]Cl (**14**).

[Ir(dfppy)$_2$-[4,4'-bis(4-(trimethylsilyl)-but-3-yn-1-yl)-2,2'-bipyridine]]Cl (15)

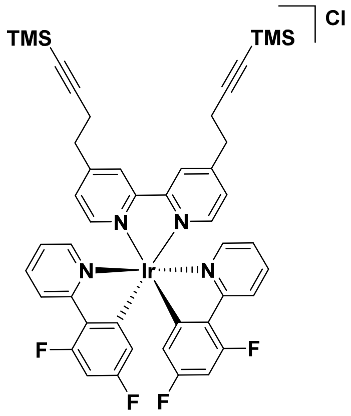

15 was synthesized following **general procedure 2**. **11** (360 mg, 0.30 mmol, 1.0 equiv.) and **6** (300 mg, 0.74 mmol, 2.5 equiv.) were added to a Schleck tube containing a mixture of DCM and MeOH (2:1, 9 mL). Ir complex **15** was obtained after column chromatography (DCM/MeOH gradient 10:0 to 10:1) as yellow solid (94 %).

^{1}H NMR (500 MHz, $CDCl_3$) δ (ppm): 10.00 (s, 2H), 8.31 (d, *J* = 8.7 Hz, 2H), 7.84 – 7.79 (m, 2H), 7.71 (d, *J* = 5.6 Hz, 2H), 7.49 (d, *J* = 5.7 Hz, 2H), 7.34 (d, *J* = 5.6 Hz, 2H), 7.05 (t, *J* = 7.2 Hz, 2H), 6.57 (ddd, *J* = 11.6, 9.0, 2.2 Hz, 2H), 5.70 (dd, *J* = 8.3, 2.3 Hz, 2H), 3.19 (ddt, *J* = 46.8, 13.7, 6.8 Hz, 4H), 2.83 (t, *J* = 6.8 Hz, 4H), -0.02 (s, 18H).

^{13}C NMR (126 MHz, CD_2Cl_2) δ (ppm): 155.83, 155.60, 154.61, 154.56, 148.54, 148.37, 138.91, 128.82, 128.30, 123.79, 123.49, 113.98, 113.84, 105.69, 99.31, 99.10, 98.89, 86.59, 33.72, 20.64, 0.03.

HRMS (NSI^+): calculated: 977.2664 [($C_{46}H_{44}F_4IrN_4Si_2$)]$^+$; measured: 977.2655.

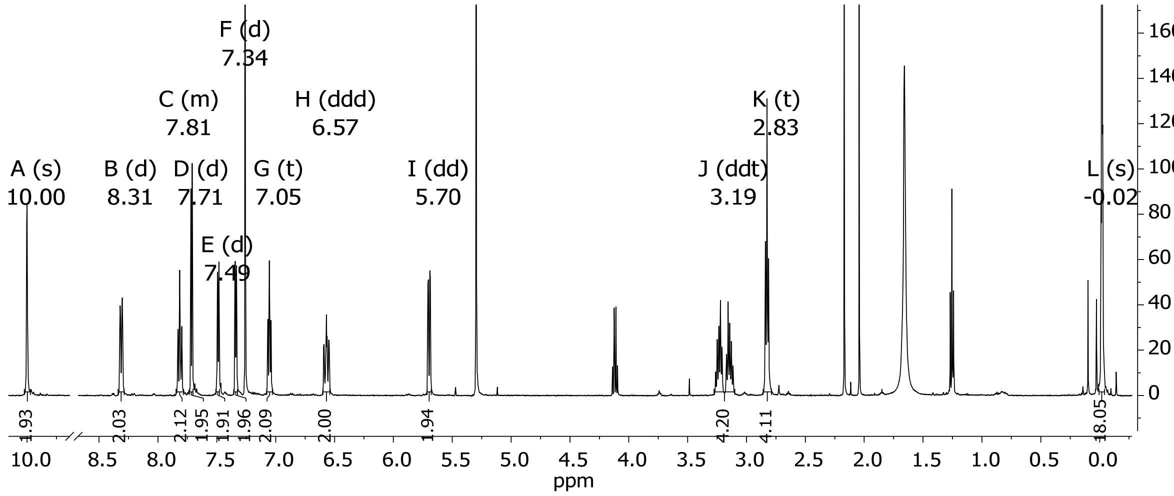

Figure S27: ^{1}H-NMR of [Ir(dfppy)$_2$-[4,4'-bis(4-(trimethylsilyl)-but-3-yn-1-yl)-2,2'-bipyridine]Cl (**15**).

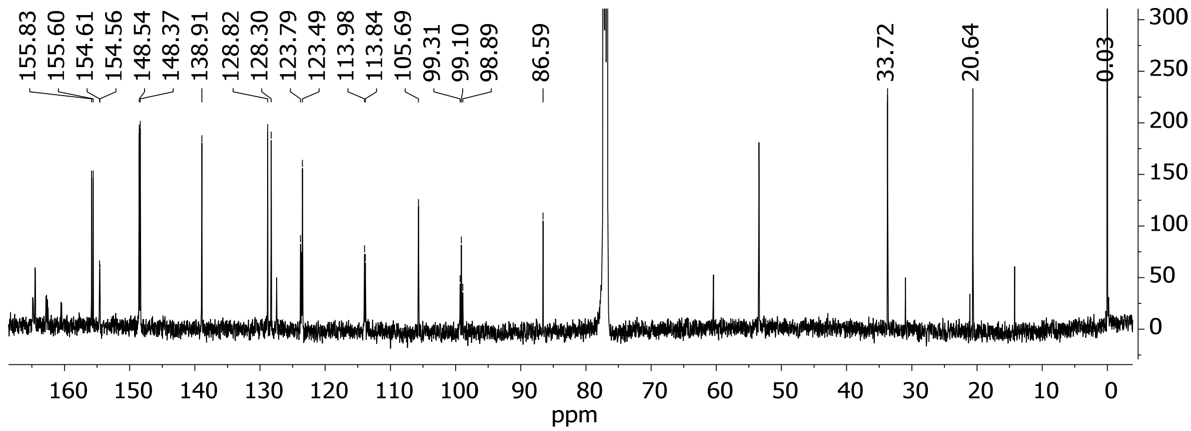

Figure S28: ^{13}C-NMR of [Ir(dfppy)$_2$-[4,4'-bis(4-(trimethylsilyl)-but-3-yn-1-yl)-2,2'-bipyridine]Cl (**15**).

General procedure 3: Synthesis of [Ir(C^N)$_2$-Ad]Cl complexes

$CuSO_4$ and L-ascorbic acid were dissolved in a mixture of H_2O and MeOH (1:1). The desired alkyne compound (1.0 equiv.) was added and the solution was vigorously stirred. After the addition of the corresponding azide (1.1/2.4 equiv.) and pyridine (5.0/10.0 equiv.), the solution was stirred for 48 hours at room temperature. The solvent was evaporated, and the product was purified by column chromatography.

[Ir(ppy)$_2$[4-(2-(1-(2-(2-(2-(2-((adamantan-1-yl)oxy)ethoxy)ethoxy)ethoxy)ethyl)-1H-1,2,3-triazol-4-yl)ethyl)-4'-methyl-2,2'-bipyridine]]Cl complex (1a)

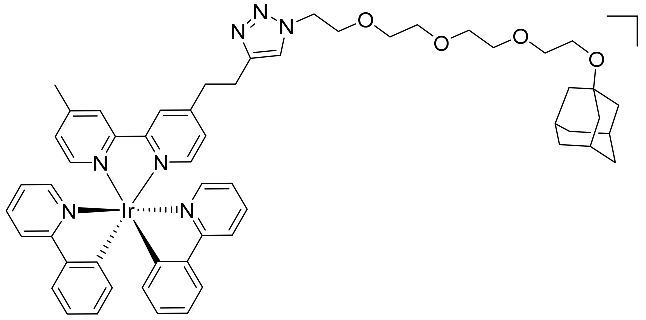

1a was synthesized following **general procedure 3**. $CuSO_4$ (3 mg, 0.019 mmol, 0.2 equiv.) and L-ascorbic acid (6.7 mg, 0.038 mmol, 0.4 equiv.) were dissolved in a mixture of H_2O and MeOH (1:1) (8 mL/8 mL). **12** (72 mg, 0.095 mmol, 1.0 equiv.) and **9** (36 mg, 0.104 mmol, 1.1 equiv.) were used to obtain yellow solid **1a** (55 mg, 4.94 mmol, 52%) by column chromatography (DCM/MeOH gradient 20:1 to 15:1).

^{1}H NMR (500 MHz, CD_2Cl_2) δ (ppm): 9.04 (d, *J* = 6.2 Hz, 2H), 8.00 – 7.96 (m, 2H), 7.93 (s, 1H), 7.86 – 7.79 (m, 4H), 7.76 (dt, *J* = 7.9, 1.6 Hz, 2H), 7.56 (dt, *J* = 5.5, 1.2 Hz, 1H), 7.49 (dt, *J* = 5.6, 1.2 Hz, 1H), 7.31 (dd, *J* = 5.6, 1.5 Hz, 1H), 7.25 (d, *J* = 5.6 Hz, 1H), 7.11 – 7.01 (m, 4H), 6.95 (tt, *J* = 7.4, 1.3 Hz, 2H), 6.34 (d, *J* = 7.7 Hz, 2H), 4.52 (t, *J* = 5.3 Hz, 2H), 3.87 (t, *J* = 5.3 Hz, 2H), 3.64 – 3.56 (m, 8H), 3.55 (s, 4H), 3.35 – 3.21 (m, 4H), 2.67 (s, 3H), 2.14 (s, 3H), 1.74 (d, *J* = 2.9 Hz, 6H), 1.70 – 1.59 (m, 6H).

^{13}C NMR (126 MHz, CD_2Cl_2) δ (ppm): 167.83/167.75, 155.77/155.71, 155.16, 152.23, 150.67/150.64, 149.87, 149.55, 148.55/148.53, 145.77, 143.77/143.72, 137.96, 131.66/131.61, 130.54/130.51, 128.73, 128.17, 126.38, 125.86, 124.79/124.76, 123.29, 123.15, 122.90/122.43, 122.41, 119.71/119.64, 71.99, 71.34, 70.49, 70.46, 70.43, 70.31, 69.44, 59.16, 50.01, 41.44, 36.40, 34.85, 30.63, 25.84, 21.21.

FTMS (NSI): m/z calculated: 1076.44 [$C_{55}H_{61}N_7O_4Ir$]$^+$, measured: 1076.44.

Anal. Calcd. for $C_{55}H_{61}ClIrN_7O_4+CH_2Cl_2$: C, 56.20; H, 5.31; N, 8.19. Found: C, 56.00; H, 5.18; N, 8.68.

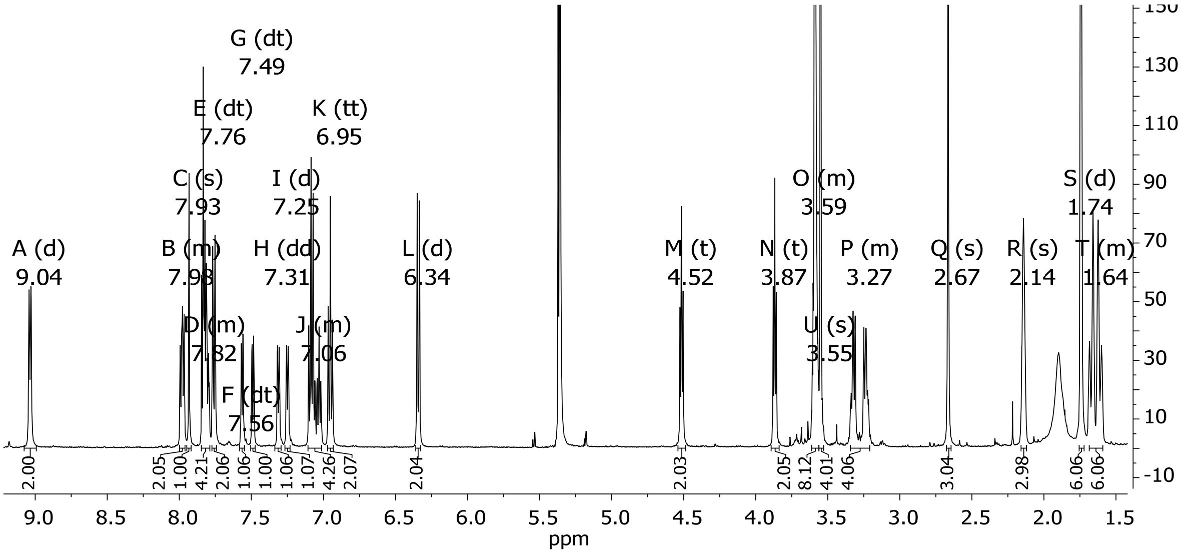

Figure S29: ^{1}H-NMR of [Ir(ppy)$_2$[4-(2-(1-(2-(2-(2-(2-((adamantan-1-yl)oxy)ethoxy)ethoxy)ethoxy)ethyl)-1H-1,2,3-triazol-4-yl)ethyl)-4'-methyl-2,2'-bipyridine]]Cl complex (**1a**).

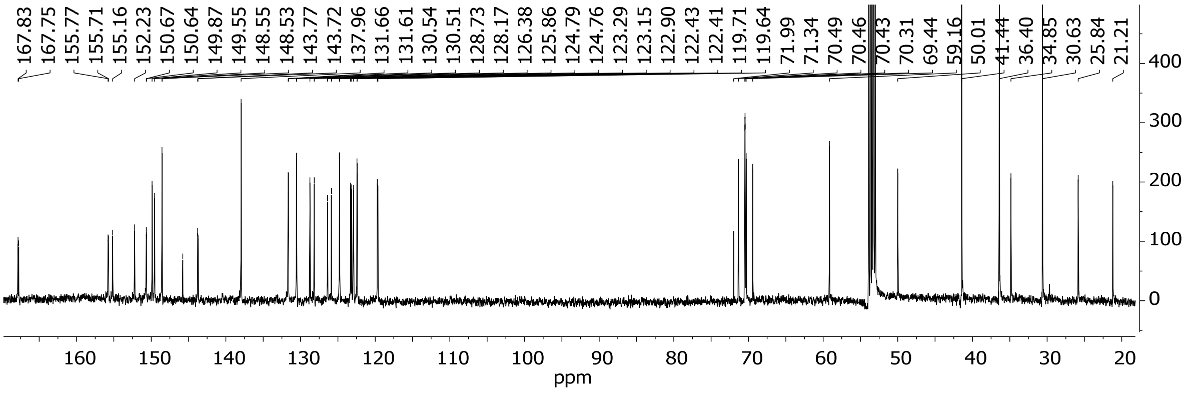

Figure S30: ^{13}C-NMR of [Ir(ppy)$_2$[4-(2-(1-(2-(2-(2-(2-((adamantan-1-yl)oxy)ethoxy)ethoxy)ethoxy)ethyl)-1H-1,2,3-triazol-4-yl)ethyl)-4'-methyl-2,2'-bipyridine]]Cl complex (**1a**).

[Ir(dfppy)$_2$[4-(2-(1-(2-(2-(2-(2-((adamantan-1-yl)oxy)ethoxy)ethoxy)ethoxy)ethyl)-1H-1,2,3-triazol-4-yl)ethyl)-4'-methyl-2,2'-bipyridine]]Cl complex (1b)

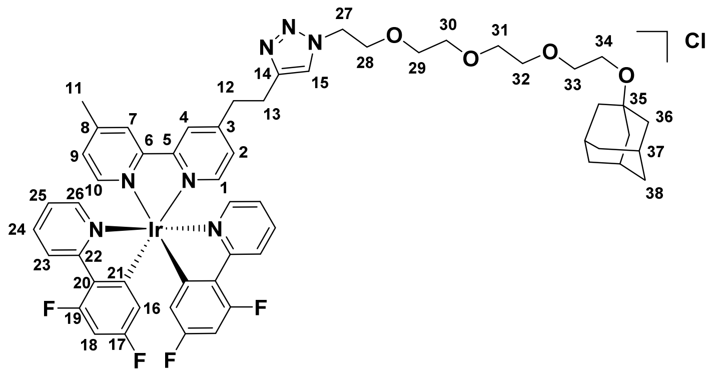

1b was synthesized following **general procedure 3**. $CuSO_4$ (26 mg, 0.16 mmol, 0.4 equiv.) and L-ascorbic acid (56 mg, 0.32 mmol, 0.8 equiv.) were dissolved in a mixture of H_2O and MeOH (1:1) (2.5/2.5 mL). **13** (350 mg, 0.40 mmol, 1.0 equiv.) and **9** (156 mg, 0.44 mmol, 1.1 equiv.) were used to obtain yellow solid **1b** (242 mg, 0.20 mmol, 50%) by column chromatography (DCM/MeOH, gradient 10:0 to 10:1).

^{1}H NMR (500 MHz, CD_2Cl_2) δ (ppm): 9.40 (s, 1H, CH-4), 9.22 (s, 1H, CH-7), 8.31 (t, *J* = 7.5 Hz, 2H, CH-23), 7.92 – 7.71 (m, 5H, CH-24, CH-1, CH-10, CH-15), 7.52 (d, *J* = 5.7 Hz, 1H, CH-26a), 7.45 (d, *J* = 5.6 Hz, 1H, CH-26b), 7.37 (d, *J* = 5.6 Hz, 1H, CH-2), 7.27 (d, *J* = 5.6 Hz, 1H, CH-9), 7.10 (t, *J* = 6.5 Hz, 1H, CH-25a), 7.04 (t, *J* = 6.6 Hz, 1H, CH-25b), 6.64 – 6.54 (m, 2H, CH-18), 5.79 – 5.70 (m, 2H, CH-16), 4.53 (s, 2H, CH_2), 3.88 (s, 2H, CH_2), 3.72 – 3.29 (m, 16H, 8xCH_2), 2.66 (s, 3H, CH_3-11), 2.11 (s, 3H, 3xCH-37), 1.71 (d, *J* = 2.9 Hz, 6H, 3xCH_2-36), 1.61 (dd, *J* = 29.9, 12.3 Hz, 6H, 3xCH_2-38).

^{13}C NMR (126 MHz, CD_2Cl_2) δ (ppm): 156.68, 156.21, 155.01, 154.96, 153.71 (C_q-3, C_q-5, C_q-6, C_q-8, C_q-22), 150.22 (CH-1), 149.84 (CH-10), 149.35 (CH-26a), 149.26 (CH-26b), 139.55 (CH-24), 129.71 (CH-9), 129.15 (CH-2), 128.27 (CH-7), 127.68 (CH-4), 124.40 (CH-23a), 124.27 (CH-23b), 124.18 (CH-25a), 124.09 (CH-25b), 114.57, 114.50, 114.45, 114.38 (CH-16a/b, C_q-21a/b), 99.60 (CH-18a), 99.39 (CH-18b), 72.54 (C_q-35), 71.91, 71.05, 70.99, 70.86, 69.77, 59.71, 51.41, 42.00 (CH_2-36), 36.96 (CH_2-38), 34.61, 31.18 (CH-37), 24.60, 21.93 (CH_3-11).

^{19}F {1 H} NMR (377 MHz, CD_2Cl_2) δ (ppm): -106.72 (d, *J* = 10.8 Hz), -106.81 (d, *J* = 10.8 Hz), -109.11 (dd, *J* = 16.6, 10.9 Hz).

HRMS (ESI): m/z calculated: 1148.4037 [$C_{55}H_{57}N_7O_4F_4Ir$]$^+$, measured: 1148.4016.

Anal. Calcd. for $C_{55}H_{57}ClF_4IrN_7O_4$+THF: C, 56.43; H, 5.22; N, 7.81. Found: C, 56.94; H, 4.86; N, 7.60.

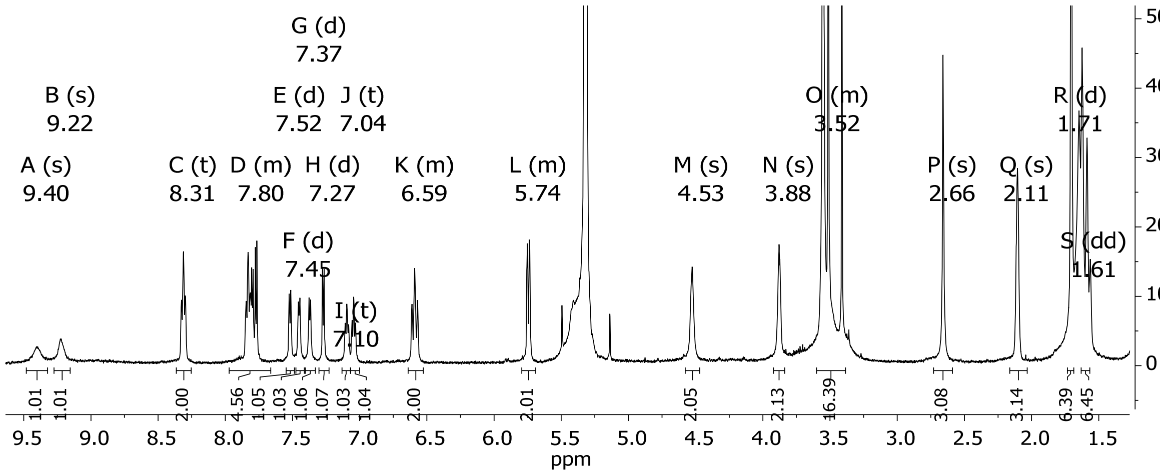

Figure S31: ^{1}H-NMR of [Ir(dfppy)$_2$[4-(2-(1-(2-(2-(2-(2-((adamantan-1-yl)oxy)ethoxy)ethoxy)ethoxy)ethyl)-1H-1,2,3-triazol-4-yl)ethyl)-4'-methyl-2,2'-bipyridine]]Cl complex (**1b**).

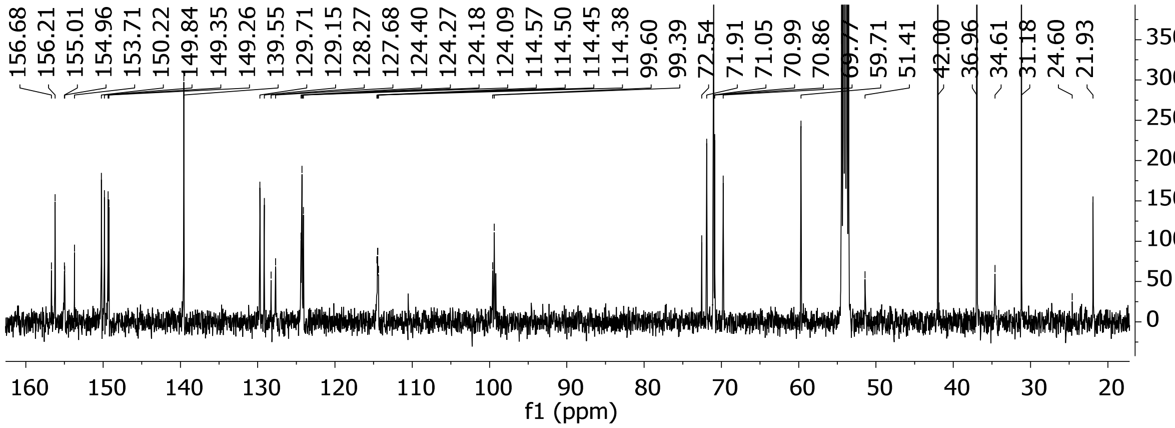

Figure S32: ^{13}C-NMR of [Ir(dfppy)$_2$[4-(2-(1-(2-(2-(2-(2-((adamantan-1-yl)oxy)ethoxy)ethoxy)ethoxy)ethyl)-1H-1,2,3-triazol-4-yl)ethyl)-4'-methyl-2,2'-bipyridine]]Cl complex (**1b**).

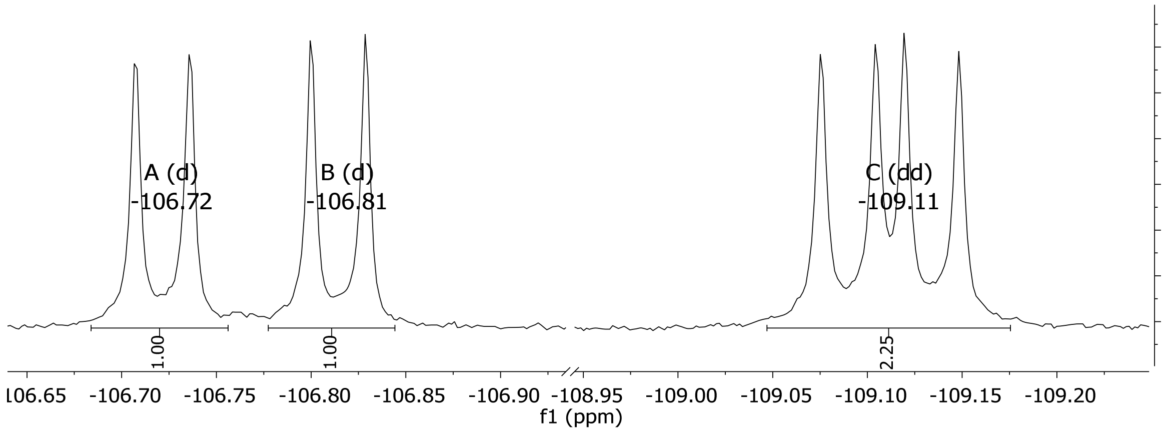

Figure S33: ^{19}F-NMR of [Ir(dfppy)$_2$[4-(2-(1-(2-(2-(2-(2-((adamantan-1-yl)oxy)ethoxy)ethoxy)ethoxy)ethyl)-1H-1,2,3-triazol-4-yl)ethyl)-4'-methyl-2,2'-bipyridine]]Cl complex (**1b**).

[Ir(ppy)$_2$-[4,4'-bis-(2-(1-(2-(2-(2-(2-((adamantan-1-yl)oxy)ethoxy)ethoxy)ethoxy)ethyl)-1H-1,2,3-triazol-4-yl)ethyl)-2,2'-bipyridine]Cl complex (2a)

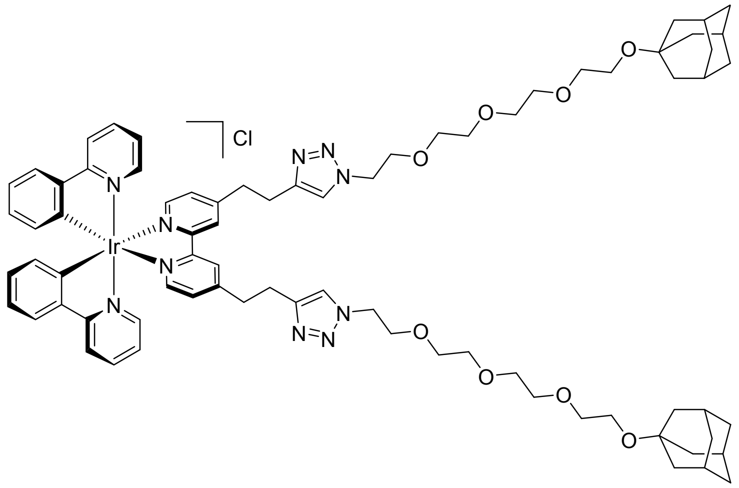

14 (360 mg, 0.38 mmol, 1.0 equiv.) was added to a round-bottom flask containing K_2CO_3 (317mg, 2.3 mmol, 5.0 equiv.) and methanol (10mL). This solution was stirred for 4 hours at room temperature. DCM and water were used for three times extractions after removing the methanol. The organic fractions were combined, washed with a portion of brine and dried over Na_2SO_4. Filtration and evaporation under reduced pressure gave the crude product. This solid was kept in vacuum for 1 hour to remove the remaining TMS, and was used for the **general procedure 3** without further purification. $CuSO_4$ (27 mg, 0.17 mmol, 0.44 equiv.) and L-ascorbic acid (60 mg, 0.30 mmol, 0.8 equiv.) were dissolved in a mixture of H_2O and MeOH (1:1) (10/10 mL) with the deprotected residue and **9** (322 mg, 0.91 mmol, 2.4 equiv.). The mixture was vigorously stirred for 48h, and the yellow solid **2a** (224 mg, 0.15 mmol, 40%) was purified by column chromatography (DCM/MeOH, gradient 10:0 to 4:1; Rf: 0.40 (5% MeOH/DCM on silica)) after removing the solvents.

^{1}H NMR (500 MHz, CD_2Cl_2) δ (ppm): 9.17 (s, 2H), 8.03 – 7.92 (m, 4H), 7.87 – 7.78 (m, 4H), 7.76 (d, *J* = 7.5 Hz, 2H), 7.52 (d, *J* = 5.2 Hz, 2H), 7.31 (d, *J* = 5.0 Hz, 2H), 7.14 – 7.03 (m, 4H), 6.95 (t, *J* = 7.2 Hz, 2H), 6.33 (d, *J* = 7.4 Hz, 2H), 4.52 (s, 4H), 3.87 (s, 4H), 3.61 – 3.54 (m, 24H), 3.38 – 3.18 (m, 8H), 2.14 (s, 6H), 1.74 (s, 12H), 1.64 (q, *J* = 11.8 Hz, 12H).

^{13}C NMR (126 MHz, CD_2Cl_2) δ (ppm): 167.75, 155.89, 155.18, 150.69, 149.81, 148.65, 143.78, 137.98, 131.61, 130.51, 128.15, 126.25, 124.77, 123.32, 122.41, 119.67, 72.05, 71.32, 70.48, 70.46, 70.42, 70.31, 69.45, 59.16, 50.13, 41.42, 36.40, 34.79, 30.63, 25.95.

HRMS (ESI): m/z calculated: 1467.6887 [$C_{76}H_{94}IrN_{10}O_8$]$^+$, measured: 1467.6840.

Anal. Calcd. for $C_{76}H_{94}ClIrN_{10}O_8$: C, 60.72; H, 6.30; N, 9.32. Found: C, 60.57; H, 6.16; N, 9.40.

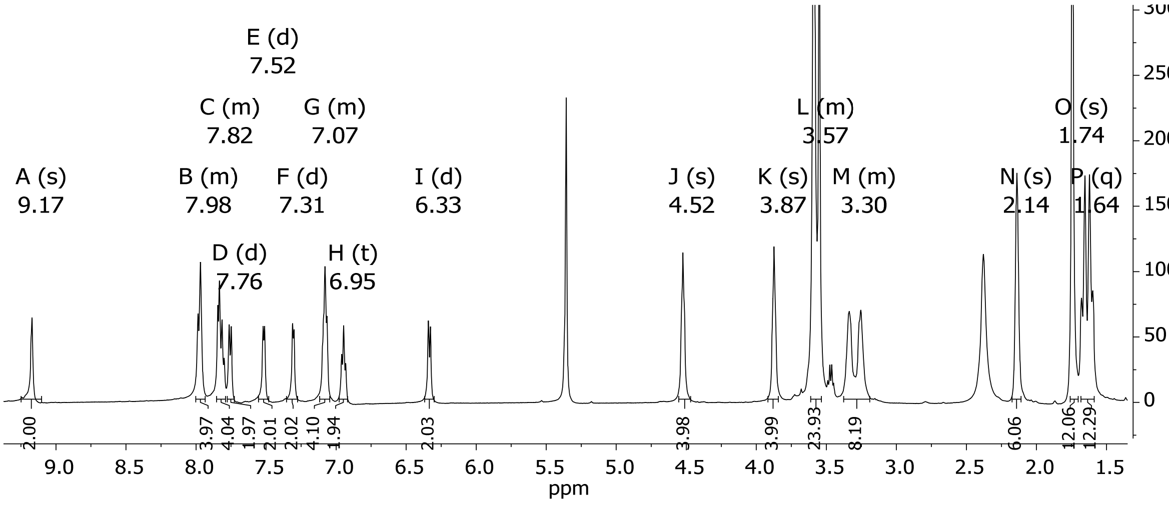

Figure S34: ^{1}H-NMR of [Ir(ppy)$_2$-[4,4'-bis-(2-(1-(2-(2-(2-(2-((adamantan-1-yl)oxy)ethoxy)ethoxy)ethoxy)ethyl)-1H-1,2,3-triazol-4-yl)ethyl)-2,2'-bipyridine]Cl complex (**2a**).

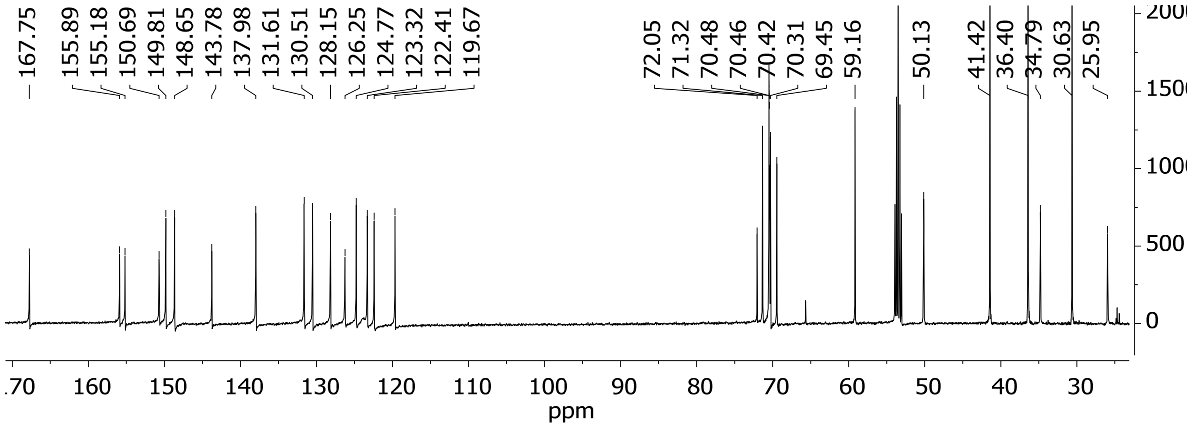

Figure S35: ^{13}C-NMR of [Ir(ppy)$_2$-[4,4'-bis-(2-(1-(2-(2-(2-(2-((adamantan-1-yl)oxy)ethoxy)ethoxy)ethoxy)ethyl)-1H-1,2,3-triazol-4-yl)ethyl)-2,2'-bipyridine]Cl complex (**2a**).

[Ir(dfppy)$_2$-[4,4'-bis-(2-(1-(2-(2-(2-(2-((adamantan-1-yl)oxy)ethoxy)ethoxy)ethoxy)ethyl)-1H-1,2,3-triazol-4-yl)ethyl)-2,2'-bipyridine]Cl complex (2b)

15 (360 mg, 0.36 mmol, 1.0 equiv.) was added to a round-bottom flask containing K_2CO_3 (317mg, 2.3 mmol, 5.0 equiv.) and methanol (10mL). This solution was stirred for 4 hours at room temperature. DCM and water were used for three times extractions after removing the methanol. The organic fractions were combined, washed with a portion of brine and dried over Na_2SO_4. Filtration and evaporation under reduced pressure gave the crude product. This solid was kept in vacuum for 1 hour to remove the remaining TMS, and was used for the **general procedure 3** without further purification. $CuSO_4$ (24 mg, 0.15 mmol, 0.4 equiv.) and L-ascorbic acid (61 mg, 0.31 mmol, 0.8 equiv.) were dissolved in a mixture of H_2O and MeOH (1:1) (10/10 mL) with the deprotected residue and **9** (297 mg, 0.84 mmol, 2.4 equiv.). The mixture was vigorously stirred for 48h, and the yellow solid **2a** (175 mg, 0.11 mmol, 30%) was purified by column chromatography (DCM/MeOH, gradient 10:0 to 10:1; Rf: 0.37 (5% MeOH/DCM on silica)) after removing the solvents.

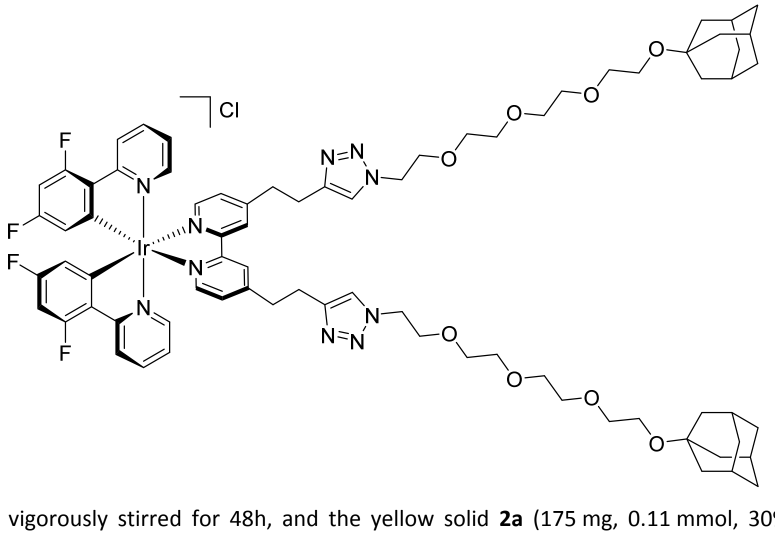

^{1}H NMR (500 MHz, CDCl$_3$) δ (ppm): 9.62 (s, 2H), 8.30 (d, *J* = 8.7 Hz, 2H), 8.01 (s, 2H), 7.81 (t, *J* = 7.8 Hz, 2H), 7.70 (d, *J* = 5.5 Hz, 2H), 7.41 (d, *J* = 5.3 Hz, 2H), 7.32 (d, *J* = 5.5 Hz, 2H), 7.09 (t, *J* = 6.3 Hz, 2H), 6.54 (t, *J* = 10.4 Hz, 2H), 5.68 – 5.61 (m, 2H), 4.48 (s, 4H), 3.84 (t, *J* = 5.2 Hz, 4H), 3.63 – 3.53 (m, 24H), 3.39 – 3.24 (m, 8H), 2.11 (s, 6H), 1.71 (s, 12H), 1.58 (q, *J* = 12.1 Hz, 12H).

^{13}C NMR (126 MHz, CDCl$_3$) δ (ppm): 156.53, 155.80, 154.57, 154.52, 149.29, 148.70, 139.07, 128.52, 127.84, 127.61, 123.93, 123.76, 123.73, 114.07, 113.93, 99.43, 99.22, 99.00, 72.41, 71.37, 70.72, 70.67, 70.65, 70.59, 69.61, 59.34, 50.14, 41.57, 36.57, 35.07, 30.60, 26.04.

HRMS (NSI): m/z calculated: 1539.65 [$C_{76}H_{90}F_4IrN_{10}O_8$]$^+$, measured: 1539.64.

Anal. Calcd. for $C_{76}H_{90}ClF_4IrN_{10}O_8$ + $CHCl_3$ + MeCN: C, 54.67; H, 5.46; N, 8.88. Found: C, 54.56; H, 4.95; N, 9.06.

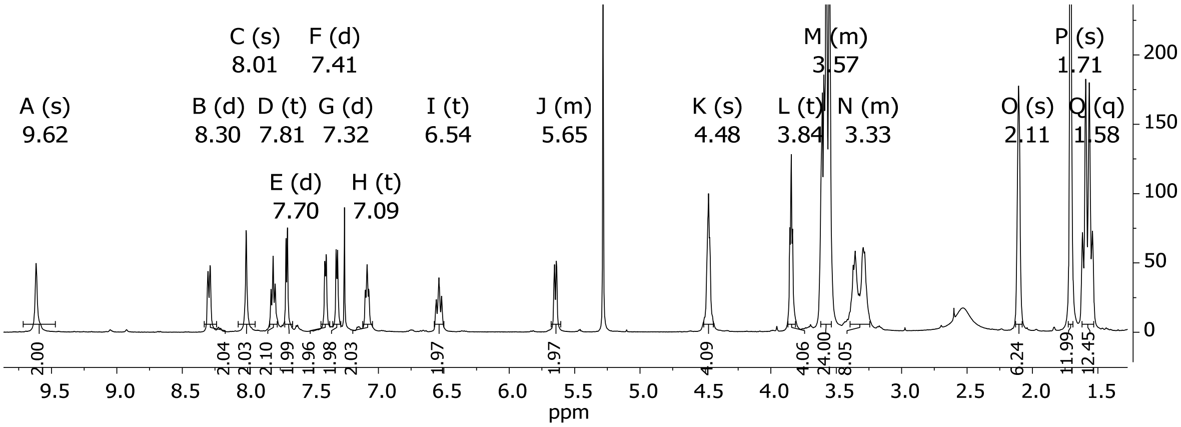

Figure S36: ^{1}H-NMR of [Ir(dfppy)$_2$-[4,4'-bis-(2-(1-(2-(2-(2-(2-((adamantan-1-yl)oxy)ethoxy)ethoxy)ethoxy)ethyl)-1H-1,2,3-triazol-4-yl)ethyl)-2,2'-bipyridine]Cl complex (**2b**).

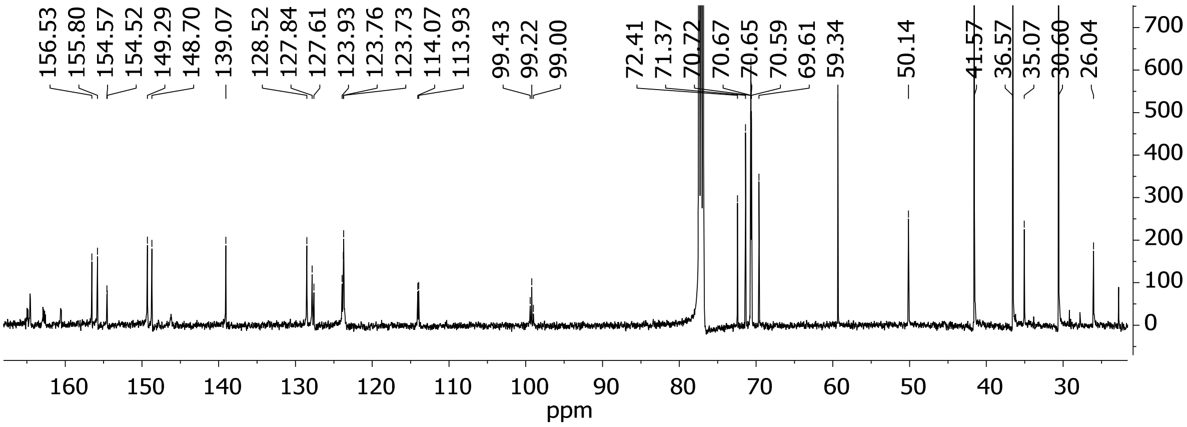

Figure S37: ^{13}C-NMR of [Ir(dfppy)$_2$-[4,4'-bis-(2-(1-(2-(2-(2-(2-((adamantan-1-yl)oxy)ethoxy)ethoxy)ethoxy)ethyl)-1H-1,2,3-triazol-4-yl)ethyl)-2,2'-bipyridine]Cl complex (**2b**).

References

1 G. A. Crosby and J. N. Demas, *J. Phys. Chem.*, 1971, **75**, 991.
2 W. H. Melhuish, *J. Phys. Chem.*, 1961, **65**, 229.
3 K. Suzuki, A. Kobayashi, S. Kaneko, K. Takehira, T. Yoshihara, H. Ishida, Y. Shiina, S. Oishi and S. Tobita, *Phys. Chem. Chem. Phys.*, 2009, **11**, 9850.
4 A. M. Brouwer, *Pure Appl. Chem.*, 2011, **83**, 2213.
5 P. Falvey, C. W. Lim, R. Darcy, T. Revermann, U. Karst, M. Giesbers, A. T. M. Marcelis, A. Lazar, A. W. Coleman, D. N. Reinhoudt and B. J. Ravoo, *Chem. Eur. J.*, 2005, **11**, 1171.
6 J. Goedhart, D. von Stetten, M. Noirclerc-Savoye, M. Lelimousin, L. Joosen, M. A. Hink, L. van Weeren, T. W. J. Gadella and A. Royant, *Nat. Commun.*, 2012, **3**, 751.
7 A. Vonderheit and A. Helenius, *PLoS Biol.*, 2005, **3**, 233.
8 J. Schindelin, I. Arganda-Carreras, E. Frise, V. Kaynig, M. Longair, T. Pietzsch, S. Preibisch, C. Rueden, S. Saalfeld, B. Schmid, J.-Y. J.-Y. Tinevez, D. J. White, V. Hartenstein, K. Eliceiri, P. Tomancak and A. Cardona, *Nat. Methods*, 2012, **9**, 676.
9 Y. Wu, M. Shi, L. Zhao, W. Feng, F. Li and C. Huang, *Biomaterials*, 2014, **35**, 5830.
10 M. C. Haberecht, M. Bolte, J. W. Bats and H. Lerner, *Zeitschirift für Naturforsch. B*, 2005, **60**, 745.
11 A. Mulder, S. Onclin, M. Péter, J. P. Hoogenboom, H. Beijleveld, J. Ter Maat, M. F. García-Parajó, B. J. Ravoo, J. Huskens, N. F. Van Hulst and D. N. Reinhoudt, *Small*, 2005, **1**, 242.
12 A. Mulder, T. Auletta, A. Sartori, S. Del Ciotto, A. Casnati, R. Ungaro, J. Huskens and D. N. Reinhoudt, *J. Am. Chem. Soc.*, 2004, **126**, 6627.
13 T. Otremba and B. J. Ravoo, *Tetrahedron*, 2017, **73**, 4972.
14 M. Nonoyama, *Bull. Chem. Soc. Jpn.*, 1974, **47**, 767.
15 A. A. Rachford, R. Ziessel, T. Bura, P. Retailleau and F. N. Castellano, *Inorg. Chem.*, 2010, **49**, 3730.
16 A. F. Henwood, A. K. Bansal, D. B. Cordes, A. M. Z. Slawin, I. D. W. Samuel and E. Zysman-Colman, *J. Mater. Chem. C*, 2016, **4**, 3726.
17 M. Mauro, K. C. Schuermann, R. Prétôt, A. Hafner, P. Mercandelli, A. Sironi and L. De Cola, *Angew. Chem. Int. Ed.*, 2010, **49**, 1222.
